# GdmRIII, a TetR Family Transcriptional Regulator, Controls Geldanamycin and Elaiophylin Biosynthesis in *Streptomyces autolyticus* CGMCC0516

**DOI:** 10.1038/s41598-017-05073-x

**Published:** 2017-07-06

**Authors:** MingXing Jiang, Min Yin, ShaoHua Wu, XiuLin Han, KaiYan Ji, MengLiang Wen, Tao Lu

**Affiliations:** Yunnan Institute of Microbiology, Yunnan University, 2 North Cui Hu Road, Kunming, Yunnan 650091 China

## Abstract

Geldanamycin and elaiophylin are co-produced in several *Streptomyces* strains. However, the regulation of their biosynthesis is not fully understood yet. Herein the function of a TetR family regulator GdmRIII, which is located in the biosynthetic gene cluster of geldanamycin, was studied to understand the regulatory mechanism of geldanamycin biosynthesis in *Streptomyces autolyticus* CGMCC0516. The production of geldanamycin decreased substantially in a Δ*gdmRIII* mutant and the yield of three compounds which were thought to be geldanamycin congeners greatly increased. Surprisingly, the structural elucidation of these compounds showed that they were elaiophylin and its analogues, which implied that GdmRIII not only played a positive regulatory role in the biosynthesis of geldanamycin, but also played a negative role in elaiophylin biosynthesis. GdmRIII affected the expression of multiple genes in both gene clusters, and directly regulated the expression of *gdmM*, *gdmN*, and *elaF* by binding to the promoter regions of these three genes. A conserved non-palindromic sequence was found among the binding sites of *elaF*. Our findings suggested that the biosynthetic pathways of geldanamycin and elaiophylin were connected through GdmRIII, which might provide a way for *Streptomyces* to coordinate the biosynthesis of these compounds for better adapting to environment changes.

## Introduction

In *Streptomyces*, the biosynthesis of natural products is under multilevel regulation that involves pathway-specific and global (pleiotropic) regulators^1–5^. Pathway-specific regulators generally regulate the biosynthesis of a specific secondary metabolite, and their genes are usually located in the respective biosynthetic gene clusters, together with genes for biosynthesis, secretion and resistance to the antibiotics. The pleiotropic regulators, which may not be linked to specific biosynthetic gene clusters, not only control the biosynthesis of secondary metabolites but also participate in other physiological processes such as cell growth and morphological differentiation. It is not yet fully understood how these multiple levels of control of secondary metabolite biosynthesis are coordinated in *Streptomyces*. Earlier investigations have suggested a paradigm that ‘low level’ pathway-specific regulators are subject to regulation by ‘higher level’ pleiotropic regulators, whose expression is typically affected by a variety of environmental and physiological cues. However, evidence also shows that cluster situated regulators thought to be pathway-specific exhibit control over ‘higher level’ regulators, and a complex network of functional interactions exists among disparate biosynthetic pathways^6^. Cross-talk also exists between global regulators^7^. Such cross-regulation further reveals the complexity and diversity of regulation that governs the biosynthesis of secondary metabolites in *Streptomyces*. It therefore presents tremendous challenges upon us to understand how environmental and developmental signals integrate in the regulatory cascades and how different levels of regulation interact with each other.

The TetR family transcriptional regulators are widely distributed among prokaryotes. Most of the TetR family regulators are associated with antibiotic resistance, antibiotic production, quorum sensing, and many other aspects of prokaryotic physiology^8–10^. The members of the TetR family are often reported to have a highly conserved helix-turn-helix DNA-binding domain and a larger C-terminal ligand-binding domain^11^. Many of these regulators bind to palindromic sequences upstream of the genes they regulate^12–14^, while others bind to non-palindromic sequences^15^. Most of the TetR family regulators are identified as homodimeric negative regulators, and only a small number act as activators^16–20^ or repressors/activators^21, 22^. Although there are more than 200,000 sequences of probable TetR family regulators in the public databases and the structure for close to 200 have been resolved, the vast majority of these regulators have not been characterized. The soil-dwelling bacteria, such as Actinobacteria, encode the highest numbers of TetR family regulators. Whole genome sequencing projects reveal that some *Streptomyces* species, including *Streptomyces coelicolor* and *Streptomyces avermitilis*, contain over 100 genes for TetR family regulators, which presumably reflects the complex morphological differentiation and secondary metabolism in these species^10^.

Benzoquinone ansamycin antibiotic geldanamycin was first found to be produced by *Streptomyces hygroscopicus* in 1970^23^, and attracted wide attention due to its antitumor activity^24, 25^. Geldanamycin is usually co-produced with another antibiotic elaiophylin^26^, which represents a class of C_2_-symmetric 16-membered macrodiolide antibiotics with various biological activities^27–31^. The fact that these two compounds are co-produced is intriguing, since they are synthesized by disparate pathways. The biosynthetic gene clusters for both compounds have been characterized^32–34^. The geldanamycin biosynthetic gene cluster contains two families of regulators, the LuxR-family regulators GdmRI and GdmRII and the TetR-family regulator GdmRIII. Meanwhile, the elaiophylin biosynthetic gene cluster contains a postulated LuxR-family regulator and two putative two-component regulators. Whether there is a connection between the geldanamycin and elaiophylin biosynthetic pathways is currently unknown.

In our previous work, a geldanamycin-producing strain *Streptomyces autolyticus* CGMCC0516 was found to be able to produce a trace of autolytimycin, whose structure is closely related to that of geldanamycin^35^. Autolytimycin has been shown to bind the heat shock protein 90 (Hsp90) with enhanced binding activity than 17-allylamino-17-demethoxygeldanamycin (17-AAG), a geldanamycin derivative currently under evaluation for treatment of cancer^36^. Since autolytimycin is a by-product of the geldanamycin biosynthetic pathway, further understanding the regulation of this pathway would bring to light new approaches for increasing the production of autolytimycin through genetic engineering. In the present work, the function of GdmRIII in *S. autolyticus* CGMCC0516 was investigated. A *gdmRIII* knockout mutant was constructed and its fermentation products were analyzed; the expression of genes in the geldanamycin and elaiophylin biosynthetic gene clusters was examined by using reverse transcription PCR (RT-PCR) and quantitative real-time PCR; GdmRIII was overexpressed in *Escherichia coli* and its binding to the potential targets was analyzed by using electrophoretic mobility shift assays (EMSAs) and DNase I footprinting assays. We found that GdmRIII played a positive regulatory role in the biosynthesis of geldanamycin, while playing a negative role in elaiophylin biosynthesis. Thus, it acted as a pleiotropic regulator in the biosynthesis of secondary metabolites in *S. autolyticus* CGMCC0516.

## Results

### GdmRIII plays a positive regulatory role in the biosynthesis of geldanamycin

**Figure 1:**
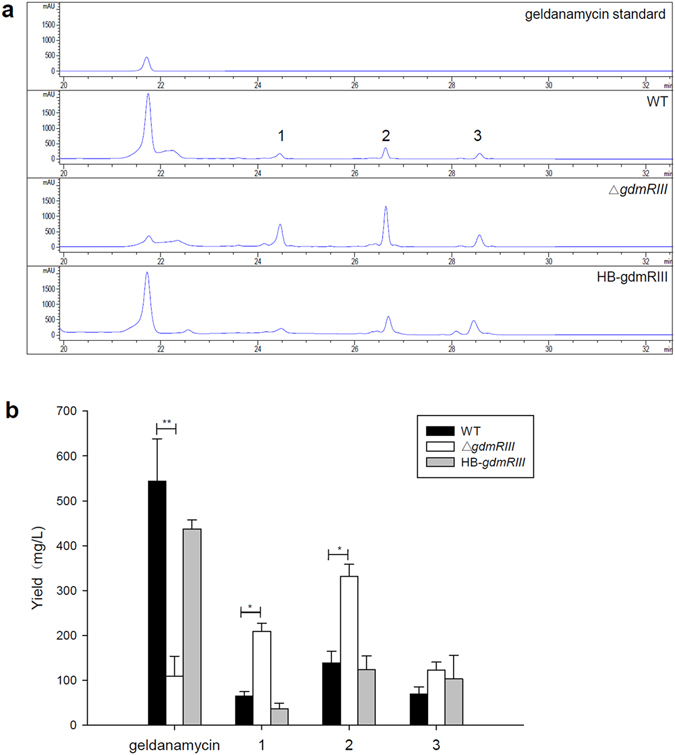
Analyses of the fermentation products of the wild-type strain, the Δ*gdmRIII* mutant, and the Δ*gdmRIII* complementation strain HB-gmdRIII. (**a**) HPLC analyses of the fermentation products; (**b**) the yield of compounds. **1**, **2**, and **3** stand for three compounds with retention time of 24.4 min, 26.6 min, and 28.4 min, respectively. Values are the average of three independent experiments, each with three replicates. Error bars indicate the standard deviation of means. The comparisons are made relative to the wild type in all instances. *p < 0.05, **p < 0.01 by paired samples t-test.

To explore the regulatory role of GdmRIII in the biosynthesis of geldanamycin, a *gdmRIII* gene knockout mutant was constructed by using the PCR-targeted gene replacement method as described^37^ (See Supplementary Fig. S1a). An Apra^R^ and Thio^S^ double-crossover Δ*gdmRIII* mutant was recovered, and verified by PCR (See Supplementary Fig. S1b) and subsequently by Southern blot (See Supplementary Fig. S1c,d). The mutant did not show any obvious morphological change or growth defect (See Supplementary Fig. S2). The fermentation products of the Δ*gdmRIII* mutant were extracted and analyzed by using HPLC. The results revealed that the production of geldanamycin substantially decreased in the mutant when compared to that in the wild-type strain (Fig. 1a). The yield of geldanamycin in the mutant was only around 19% of that in the wild-type strain on day 7 (Fig. 1b). Meanwhile, the yields of three compounds with retention time of 24.4 min (compound **1**), 26.6 min (compound **2**), and 28.4 min (compound **3**) increased to about 3.2 fold, 2.4 fold, and 1.7 fold of that in the wild-type strain, respectively (Fig. 1a,b). To exclude the polar effect of the replacement cassette on the nearby genes in the cluster, the wild-type *gdmRIII* gene was cloned in pUWL201apr, in which the expression of *gdmRIII* was under the control of a constitutive promoter ermE*, and introduced into the Δ*gdmRIII* mutant. The complementation analysis showed that the production of geldanamycin recovered to 80% of the regular level after introduction of a wild-type *gdmRIII* gene into the mutant. At the same time, the yields of the other three compounds almost returned to the levels of that in the wild-type strain (Fig. 1a,b), indicating that the changes of the production of these compounds were due to the mutation in *gdmRIII*. Thus, GdmRIII played a positive regulatory role in the biosynthesis of geldanamycin, which was consistent with the published report that a homologue of GdmRIII, named Gel19, positively control the biosynthesis of geldanamycin in *S. hygroscopicus* JCM4427^38^.

### The secondary metabolites whose production increased in the Δ*gdmRIII* mutant are elaiophylin and its analogues

Our previous experiments showed that the production of three compounds, **1**, **2**, and **3**, increased in the Δ*gdmRIII* mutant. We were interested in whether these compounds were intermediates of the geldanamycin biosynthetic pathway. Fermentation with the Δ*gdmRIII* mutant was carried out for 7 days, and compound **1**, **2**, and **3** were purified and their chemical structures were determined. Surprisingly, the ESI-MS analyses showed that the molecular weight of compound **1**, **2**, and **3** were 1024, 1038 and 1052, based on *m/z* 1023 [M – H]^−^, 1061 [M + Na]^+^, and 1051 [M – H]^−^, respectively. ^1^H-NMR, ^13^C-DEPT NMR, HSQC, HMBC, ^1^H-^1^H COSY, and ROESY analyses indicated that they were elaiophylin, 11′-*O*-methylelaiophylin, and 11, 11′-*O*-dimethylelaiophylin, respectively (see Supplementary Figs S3–S24 and Tables S1–S3). The spectra were consistent with those in the literature^28, 29^. Therefore, GdmRIII also regulated the elaiophylin biosynthetic pathway, in a negative way. This was unexpected, since there is no report yet showing that GdmRIII, a member of the TetR family regulators situated in the geldanamycin gene cluster, could control the biosynthesis of elaiophylin.

### GdmRIII positively regulates the expression of multiple genes in the geldanamycin gene cluster

**Figure 2:**
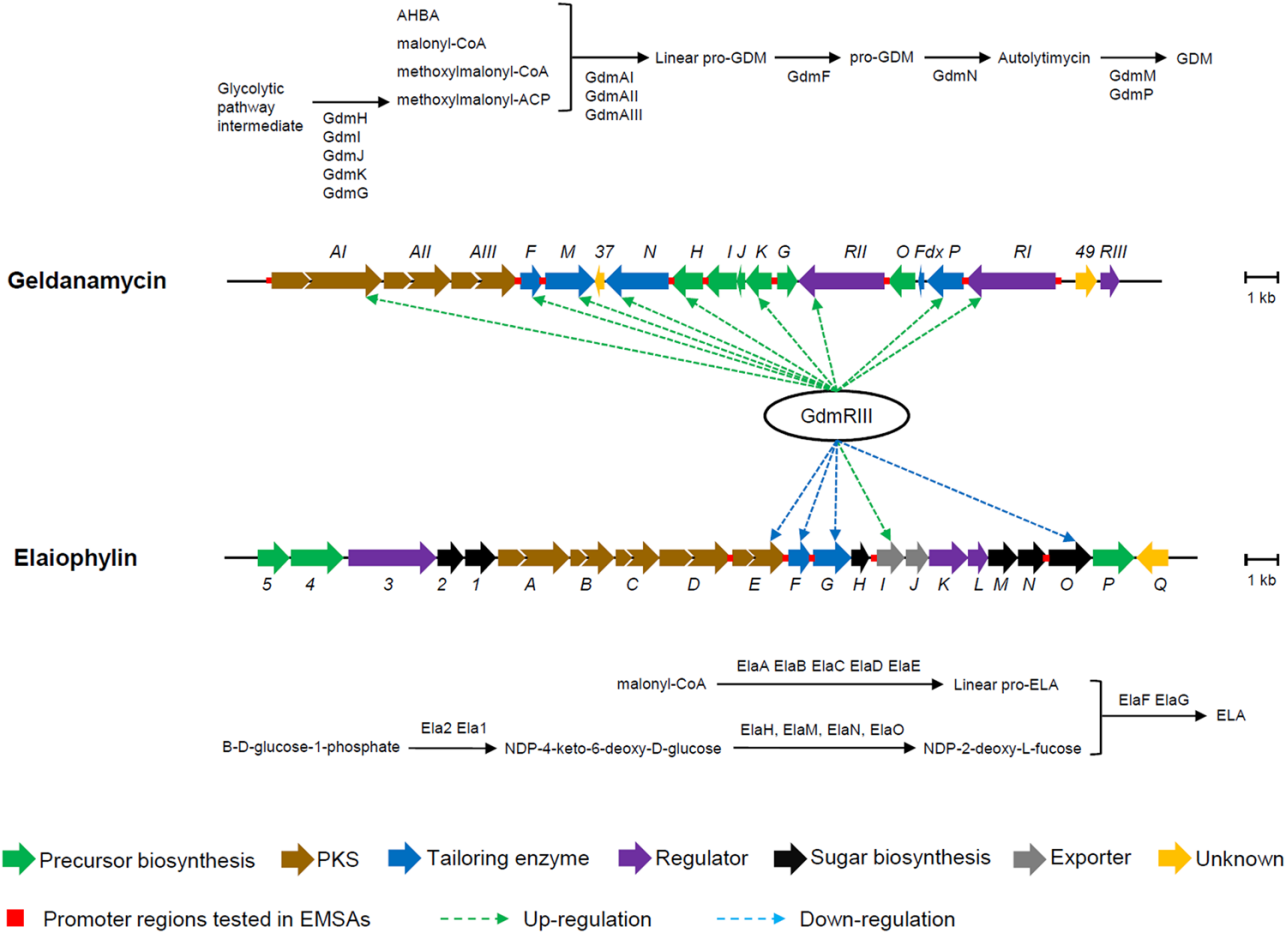
The geldanamycin (GDM) and the elaiophylin (ELA) gene clusters from *S. autolyticus* CGMCC0516 unveiling the genes regulated by GdmRIII and the steps that these genes participate in their respective biosynthetic pathways.

**Figure 3:**
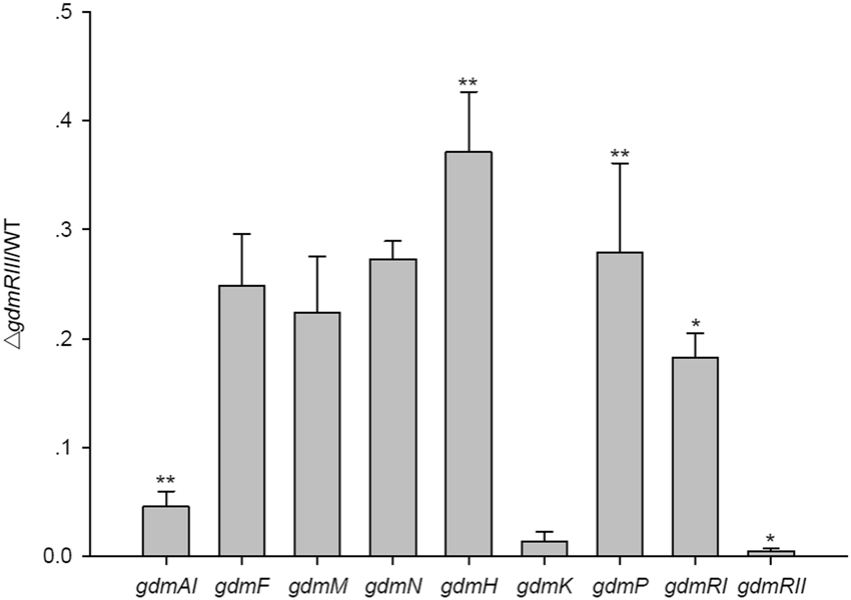
Analyses of the transcriptional levels of genes in the geldanamycin biosynthetic gene cluster. Values are the average of three independent experiments, each with three replicates. Error bars indicate the standard deviation of means. *p < 0.05, **p < 0.01 by paired samples t-test.

We then tried to identify the genes whose expression was under the control of GdmRIII in the geldanamycin biosynthetic gene cluster by comparing their transcriptional levels in the Δ*gdmRIII* mutant to that in the wild-type strain. The transcription of genes involved in the biosynthesis of geldanamycin, including those encoding polyketide synthases (PKSs), tailoring enzymes, and regulators (the genetic organization of the geldanamycin gene clusters is illustrated in Fig. 2), was examined by using RT-PCR. The results showed that the transcription of genes involved in multiple processes of geldanamycin biosynthesis was affected by the Δ*gdmRIII* mutation. Specifically, the transcriptional levels of PKS gene *gdmAI*, tailoring enzyme genes *gdmF*, *gdmM*, *gdmN*, *gdmH*, *gdmK*, and *gdmP*, and regulatory genes *gdmRI* and *gdmRII* in the Δ*gdmRIII* mutant decreased or were abolished when compared to that in the wild-type strain on day 5 (See Supplementary Fig. S25). Quantitative real-time PCR was then used to further confirm the changes of the transcriptional levels of these genes, and the results showed that the transcription of *gdmAI*, *gdmF*, *gdmM*, *gdmN, gdmH, gdmK, gdmP, gdmRI*, and *gdmRII* indeed decreased in the Δ*gdmRIII* mutant (Fig. 3). Therefore, GdmRIII controlled, directly or indirectly, the expression of multiple genes in the geldanamycin biosynthetic pathway (illustrated in Fig. 2).

### GdmRIII negatively regulates the expression of multiple genes in the elaiophylin gene cluster

**Figure 4:**
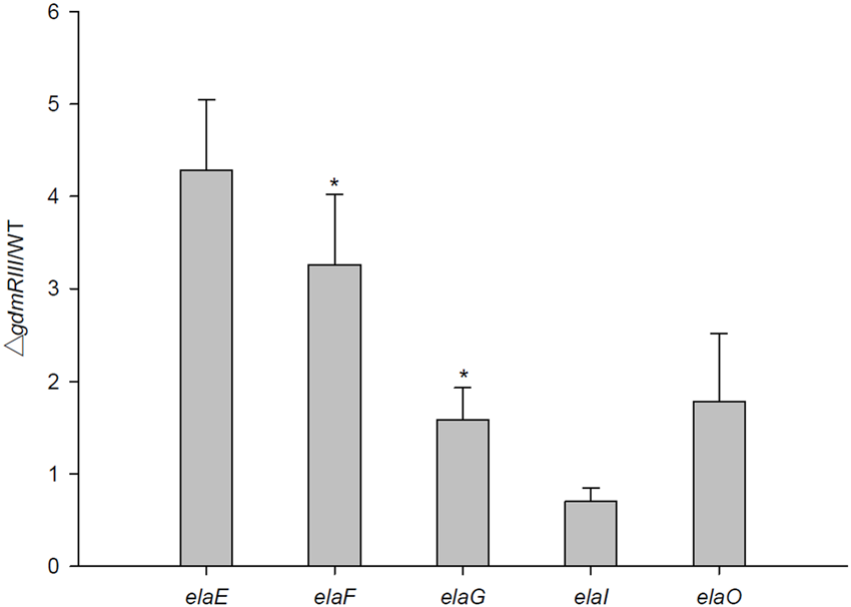
Analyses of the transcriptional levels of genes in the elaiophylin biosynthetic gene cluster. Values are the average of three independent experiments, each with three replicates. Error bars indicate the standard deviation of means. *p < 0.05, **p < 0.01 by paired samples t-test.

To find out whether GdmRIII negatively regulates the expression of genes in the elaiophylin gene cluster, RT-PCR was first carried out to examine the transcriptional levels of six major genes in the cluster, *elaE*, *elaF*, *elaG*, *elaI*, and *elaO* (the genetic organization of the elaiophylin clusters is illustrated in Fig. 2). The results showed that the transcription of *elaE*, *elaF*, *elaG*, and *elaO* were enhanced in the Δ*gdmRIII* mutant on day 5 compared to that in the wild-type strain (See Supplementary Fig. S26). Therefore, the expression of these genes might be negatively controlled by GdmRIII (illustrated in Fig. 2). However, the transcription of *elaI* was a little weakened in the Δ*gdmRIII* mutant. Quantitative real-time PCR was then performed to further verify the effect of the Δ*gdmRIII* mutation on the expression of above genes. The results confirmed that the transcription of genes *elaE*, *elaF*, *elaG*, and *elaO* all increased in the mutant compared to that in the wild-type strain, while the transcription of *elaI*, which encodes for an exporter, decreased slightly (Fig. 4). The increase of expression of the major genes related to elaiophylin biosynthesis was correlated with the yield increase of elaiophylin and its analogues in the Δ*gdmRIII* mutant. Therefore, GdmRIII regulated two disparate biosynthetic pathways of secondary metabolites in *S. autolyticus* CGMCC0516, in an antagonistic mode.

### GdmRIII binds to the promoter regions of genes *gdmM*, *gdmN*, and *elaF in vitro*

To examine whether GdmRIII binds to the promoter regions of genes whose expression was affected by GdmRIII to directly regulate their expression, GdmRIII protein was overexpressed in *E. coli* and purified. The binding of GdmRIII to the probable promoters of genes in the geldanamycin and elaiophylin gene clusters was then examined by using EMSAs. SDS-PAGE analysis of the purified GdmRIII showed that the molecular weight of His_6_GdmRIII was 27.9 kDa as anticipated (See Supplementary Fig. S27a). The purified GdmRIII was further verified by western blot (See Supplementary Fig. S27b).

**Figure 5:**
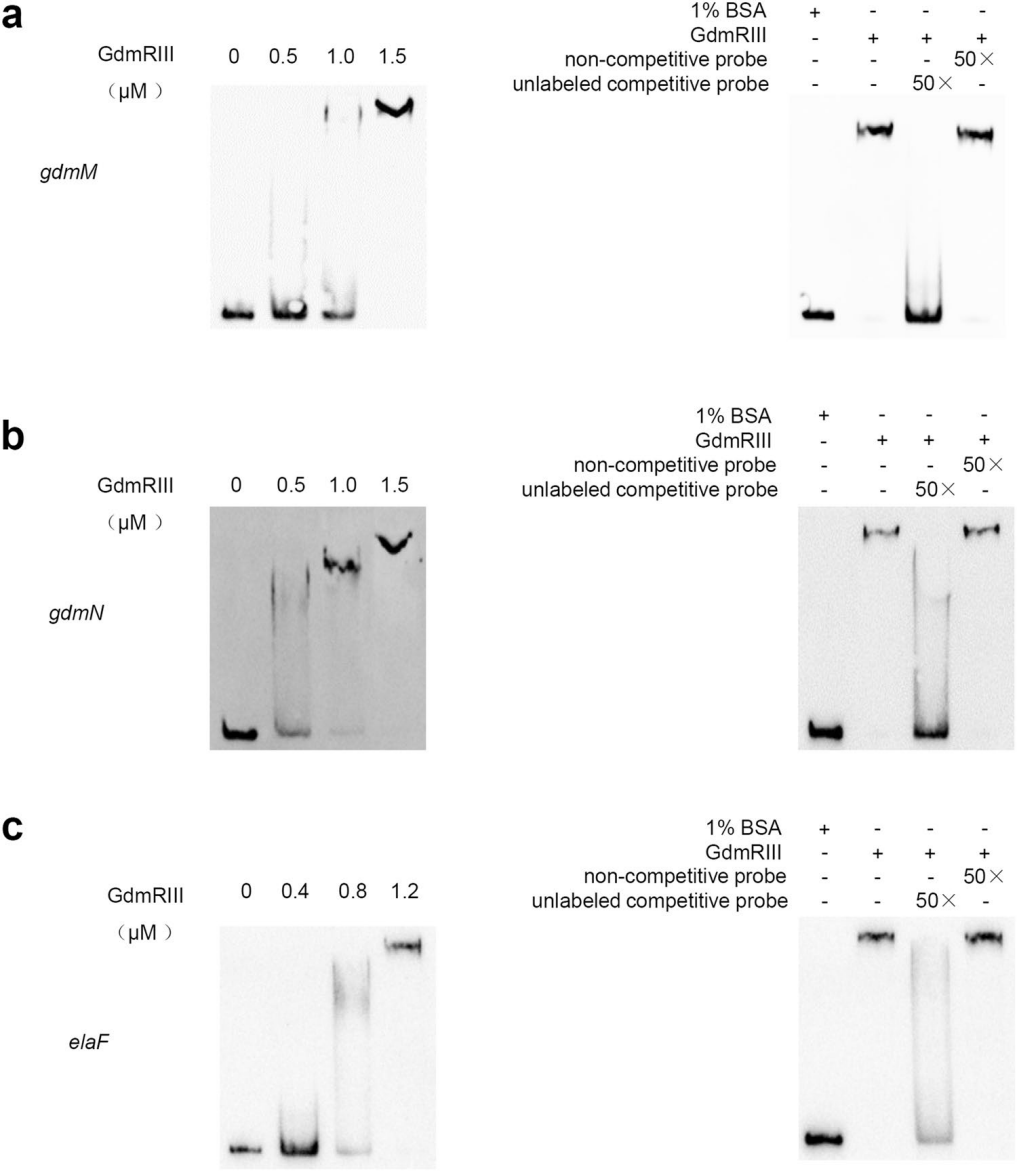
EMSA analyses of the binding of GdmRIII to its targets in the geldanamycin and elaiophylin gene clusters. The binding of GdmRIII, with gradually increased protein concentrations, to the promoter regions of *gdmM* (**a**), *gdmN* (**b**), and *elaF* (**c**) are shown. For controls, 1% BSA was used instead. The concentrations of GdmRIII used in the competitive EMSAs were 1.5 μM for *gdmM*, *gdmN* and 1.2 μM for *elaF*. The concentration of promoter sequences used in the normal or the competitive EMSAs was 0.3 nM. The experiments were repeated three times, and the representative images are shown.

The binding of GdmRIII to the probable promoter regions of genes in the geldanamycin and elaiophylin gene clusters was then analyzed. The upstream regions of genes *gdmAI*, *gdmF*, *gdmM*, *gdmN*, *gdmH*, *gdmK*, *gdmP*, *gdmRI*, and *gdmRII* in the geldanamycin cluster and *elaE*, *elaF*, *elaG*, *elaI*, and *elaO* in the elaiophylin cluster (illustrated in Fig. 2) were amplified from the genomic DNA of the wild-type strain. EMSAs were carried out, and the results showed that GdmRIII could bind to the promoter regions of *gdmM*, *gdmN*, and *elaF in vitro* (Fig. 5). Competitive EMSAs indicated that the binding could be easily competed by 50-fold of specific DNA, however not by 50-fold of nonspecific DNA. The binding of GdmRIII to the promoter regions of these genes further confirmed the direct regulatory role of GdmRIII on them. However, no binding of GdmRIII protein to the promoter regions of other genes was found, which might be due to the non-optimal conditions used in EMSAs, lack of necessary cellular factors, or the possibility that their expression was not under the direct control of GdmRIII.

### GdmRIII binds to a conserved non-palindromic sequence

**Figure 6:**
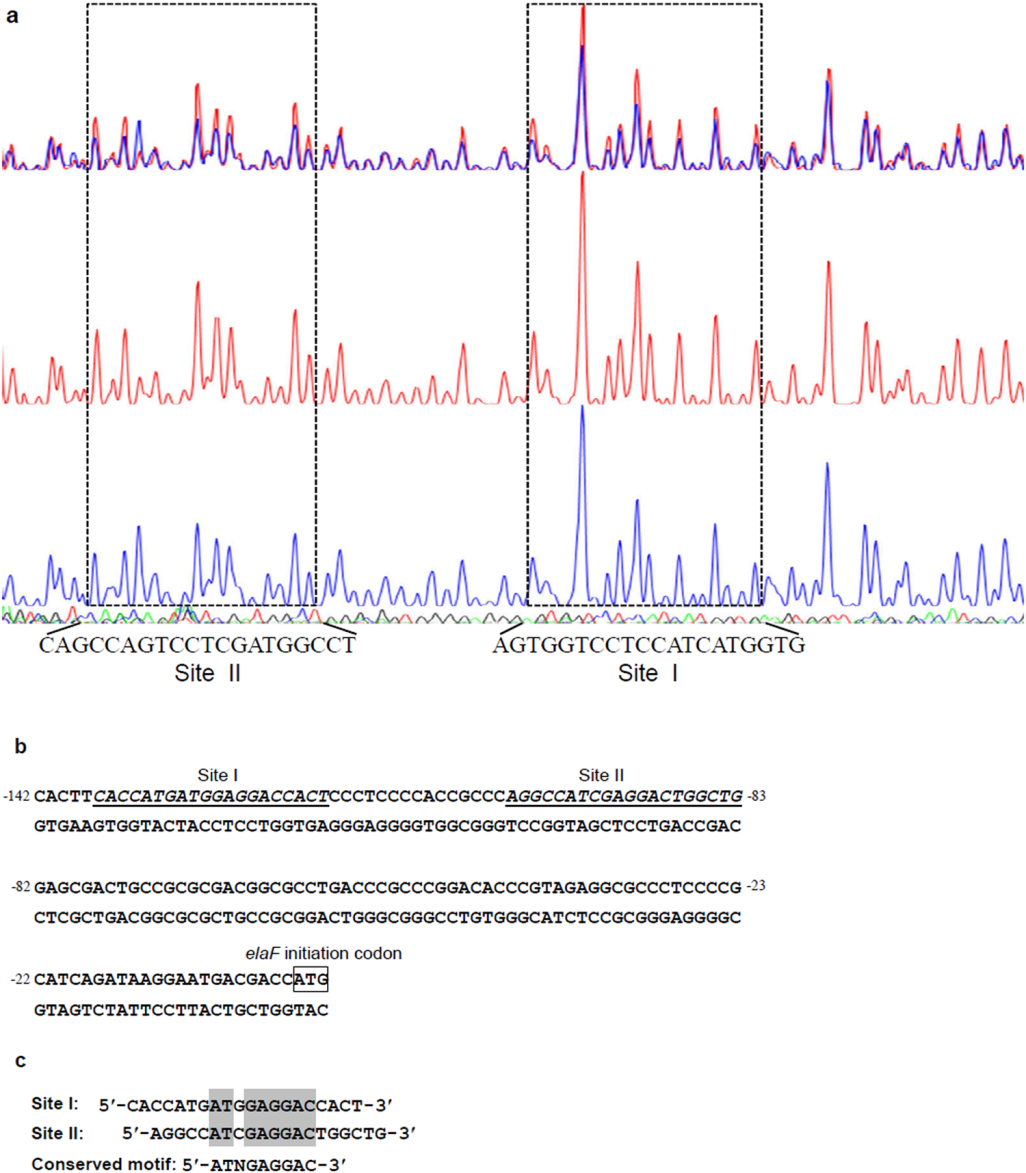
Determination of the binding sites of of GdmRIII. (**a**) DNase I footprinting assay of the *elaF* promoter protected by GdmRIII. The middle electropherogram represents the sequencing reaction without GdmRIII protein and the lower electropherogram represents the reaction with GdmRIII protein added. The regions where the blue peaks are constantly lower than the red peaks are considered to be protected by GdmRIII; (**b**) the nucleotide sequence of the *elaF* promoter region and the GdmRIII binding sites. The numbers indicate the distance (nt) from the translational start point. The solid lines indicate the GdmRIII binding sites. (**c**) the conserved sequence among GdmRIII binding sites.

In order to precisely pinpoint the binding sites of GdmRIII, DNase I footprinting assays were performed to identify the promoter sequence of *elaF* protected by GdmRIII. The results showed that the regions protected by GdmRIII were −137 to −118 nt (5′-CACCATGATGGAGGACCACT-3′) and −102 to −83 nt (5′-AGGCCATCGAGGACTGGCTG-3′) relative to the translation start codon of *elaF* (Fig. 6). The changes did not look very significant, which might be caused by weak binding of GdmRIII to its targets under the conditions used for DNase I footprinting. A conserved sequence (5′-ATNGAGGAC-3′) was found among the binding sites. However, there was no apparent palindromic sequence in the binding sites, which is often recognized by TetR family regulators^8^. Nevertheless, our results demonstrated that GdmRIII could directly bind to the promoter regions of genes in the geldanamycin and elaiophylin gene clusters to regulate their expression, and provided another example of the diversity among operator sequences of the TetR family regulators, which are often difficult to accurately predict^8^.

## Discussion

In the present work, the TetR family regulator GdmRIII was found to not only control the biosynthesis of geldanamycin but also regulate the biosynthesis of elaiophylin and its analogues, acting as a pleiotropic regulator in the biosynthesis of bioactive secondary metabolites in *S. autolyticus* CGMCC0516.

A homologous protein of GdmRIII (named Gel19) is reported as a positive regulator in the biosynthesis of geldanamycin in *S. hygroscopicus* JCM4427^38^, which was confirmed by our analyses. However, the Δ*gdmRIII* mutant of *S. autolyticus* still produced geldanamycin, albeit substantially less, unlike that of *S. hygroscopicus* JCM4427, where production of geldanamycin is totally abolished. Accordingly, the expression of *gdmAI* was detected, though greatly decreased, in the Δ*gdmRIII* mutant but not in the Δ*gel19* mutant. Furthermore, the expression of post-modification gene *gdmN* (*gel8*) and the LuxR regulator gene *gdmRI* (*gel17*) decreased in the Δ*gdmRIII* mutant, contrary to that reported in *S. hygroscopicus* JCM4427. The discrepancy might be due to the different background of strains used. The expression of other genes, such as *gdmP* (*gel16*) and *gdmRII* (*gel14*), either decreased or was abolished, which was consistent with that reported^38^. In addition, we found that the expression of some other biosynthetic genes, *gdmF*, *gdmM*, *gdmH*, and *gdmK*, were all affected by the Δ*gdmRIII* mutation. Their expression is not examined in the reported work^38^. The effect of the Δ*gdmRIII* mutation on these genes might be indirect, since the mutation caused decreases in the transcriptional levels of two LuxR-family regulator genes, *gdmRI* and *gdmRII*, which are known to positively control the expression of polyketide biosynthetic related genes^38, 39^.

The intergenic regions between *gdmRIII* and adjacent genes are less than 200 bp, which is consistent with that in most of the TetR regulators. The DNase I footprinting assays showed that GdmRIII bound to two sites in the promoter region of *elaF*. No palindromic motifs were found in the binding sites, which is also observed with another TetR family regulator AtrA that regulates the biosynthesis of daptomycin in *Streptomyces roseosporus* ^15^. This further illustrated the reported diversity of operator sequences of TetR family regulators^10^.

The elaiophylin gene cluster and its biosynthetic pathway are previously reported^34^, however little is known about the regulation of this pathway. In our work, GdmRIII was found also regulating the biosynthesis of elaiophylin. Our results showed that the transcriptional levels of related genes *elaE* (Type I PKS), *elaF* (Type II thioesterase), *elaG* (glycosytransferase), and *elaO* (NDP-hexose 2,3-dehydratase) increased in the Δ*gdmRIII* mutant when compared to that in the wild-type strain, suggesting GdmRIII negatively controlled their expression. The EMSA analyses indicated that GdmRIII bound to the promoter region of *elaF*, therefore GdmRIII might directly control the expression of *elaF*, whose function is proposed to unblock PKS modules and restore overall efficiency of the complex enzyme^40^. To the best of our knowledge, this is the first report that the biosynthesis of elaiophylin is under the control of GdmRIII, which resides within the gene cluster of geldanamycin biosynthesis.

Elaiophylin is usually co-produced with geldanamycin^26^, but whether there is an underlying relationship between the two pathways is unknown. The fact that GdmRIII simultaneously regulated these two pathways in *S. autolyticus* CGMCC0516 suggested that the two pathways were actually connected, at least partially, through GdmRIII. The control of GdmRIII over these two disparate pathways is interesting, since being a cluster situated regulator, GdmRIII was thought to be pathway-specific, but not pleiotropic. The cross-regulation of disparate biosynthetic pathways we found with GdmRIII, resonated with a few other reports^6, 16, 41–43^, further revealed the complexity of regulation that governs the biosynthesis of secondary metabolites in *Streptomyces*. It has long been recognized that the production of multiple secondary metabolites is coordinated during the growth cycle of *Streptomyces*, perhaps to facilitate their ability to compete against other biological species^44^. While the biological significance of cross-regulation exerted by GdmRIII is unknown, it might be a way for cells to coordinate disparate biosynthetic pathways for better adapting to environmental changes.

GdmRIII controlling the biosynthetic pathways of geldanamycin and elaiophylin in an antagonistic mode was rather intriguing. A similar regulatory pattern is also observed with RrdA in *S. coelicolor*, in which an *rrdA* null mutant exhibits increased undecylprodigiosin production and decreased actinorhodin production^45^. However, different from that with GdmRIII, the gene encoding RrdA resides within neither of these two gene clusters, and its influence on the biosynthesis of actinorhodin is supposed to be exerted at a non-transcriptional level. Biosynthesis of secondary metabolites is highly regulated in *Streptomyces*, which is associated with specific stages of the growth cycle, and often occurs during nutrient limitation. The supply of metabolites utilized in common by different pathways has been postulated to alter the actions of pathways whose protein products compete for the same precursor^46^. Since malonyl-CoA and methylmalonyl-CoA are both used as the extension units for the biosynthesis of geldanamycin^47^ and elaiophylin^48^, the competition between these two pathways for pools of precursor metabolites used in common might be the reason they were regulated in an antagonistic mode, which in turn might confer an adaptive advantage by selectively synthesizing the needed compound during nutrient limitation. It is worth noting that the presence or lack of GdmRIII did not completely shut down the production of elaiophylin or geldanamycin, which is consistent with the fact that these two compounds are usually co-produced. Other unknown cellular factors must have dictated the biosynthesis of geldanamycin and elaiophylin, while GdmRIII works to fine tune their relative amount to exert more elaborate control over the processes. Further studies on the distinct role of GdmRIII played in disparate biosynthetic pathways would help us to understand the complex control of secondary metabolism in *Streptomyces*.

## Methods

### Strains, plasmids, and growth conditions

Wild-type strain *S. autolyticus* CGMCC0516 has been described previously^35^. *Escherichia coli* BW25113/pIJ790^37^ was used for PCR-targeting and λ-RED-mediated mutagenesis, ET12567/pUZ8002^49^ for intergeneric conjugation, DH5α^50^ for routine cloning, and BL21(DE3)^51^ for protein expression. Cosmid pSA2065, which contains the wild-type *gdmRIII* gene, was used for constructing the gene knockout. pHY773, a derivative of pIJ773^37^ in which the *oriT* fragment has been deleted, was used as the template for amplifying the replacement cassette. pUWL201apr, which contains the constitutive promoter ermE* upstream of the multiple cloning site, was used for constructing the complementation plasmid. pEASYT1 (Trans, Beijing) was used as the T-cloning vector, and pET29a (Novagen, Madison) for protein expression.

*E. coli* strains were grown at 37 °C in Luria-Bertani (LB) medium or on LB agar plates supplemented with appropriate antibiotics^50^. *Streptomyces* strains were grown at 28 °C on ISP4 (BD, Franklin Lakes) to obtain spores, in TSB (BD) for seeds (cultures used to inoculate the media for fermentation), and in 61# medium (soya bean powder 5 g, Fish peptone 2 g, glucose 20 g, soluble starch 5 g, yeast extract 2 g, NaCl 4 g, K_2_HPO_4_ 0.5 g, MgSO_4_·7H_2_O 0.5 g and CaCO_3_ 2 g in 1 liter tap water, pH 7.8) for fermentation. IWL4 solid medium (ISP4 medium supplemented with 0.05% yeast extract, 0.1% tryptone, and 20 mM MgCl_2_) was used to plate out conjugation mixtures. Apramycin (50 µg/mL), chloramphenicol (50 µg/mL), kanamycin (50 µg/mL), ampicillin (100 µg/mL), thiostrepton (50 µg/mL), and nalidixic acid (100 µg/mL) were added to the medium as required.

### Gene disruption and complementation

DNA isolation and manipulation in *E. coli* and *Streptomyces* were carried out according to standard procedures^49, 50^. A Δ*gdmRIII* mutant strain was constructed as described^37^. The *gdmRIII* gene disruption cassette was PCR amplified from pHY773 with primers 5′-TATTGGCTGACAGCCAGCCAACGCAGGAGTTACAGCATGATTCCGGGGATCCGTCGACC-3′ and 5′-GGGCCCGTCCCTCACCCATGAGTCACCTCTGAGTGCTCATGTAGGCTGGAGCTGCTTC-3′, and introduced into BW25113/pIJ790/pSA2065 by electroporation. The strain harboring the disrupted *gdmRIII* gene on pSA2065 was screened on LB plates supplemented with apramycin. The resultant cosmid, named pSRIII, was introduced into ET12567/pUZ8002 by electroporation, and then conjugation between ET12567/pUZ8002/pSRIII and *S. autolyticus* CGMCC0516 was carried out as described^49^. The double-crossover mutants were identified by screening for Apra^R^ and Thio^S^ colonies. The mutants were verified by PCR amplification with primers 5′-GGAGCATCTCCCGGTGGACCG-3′ and 5′-CCGCTCTCCGGCGACACGCTCA-3′, and by Southern blot^50^ using a DIG High Prime DNA Labeling and Detection Starter Kit I (Roche, Basel).

For complementation analysis, *gdmRIII* was amplified from wild-type *S. autolyticus* by using primers 5′-CCCAAGCTTATGGTCCCCCGAAGCCCGTCGGTCAAT-3′ and 5′-GGAATTCTCATCGACTCCCGTCGGGCGGC-3′. The amplicon was cloned into pUWL201apr and then introduced into the Δ*gdmRIII* mutant by conjugation to obtain the Amp^R^ Thio^R^ complementation strain HB-gdmRIII.

### Fermentation and HPLC analysis of compounds

*Streptomyces* strains were grown on ISP4 plates at 28 °C for 72 hr, inoculated into TSB medium, and further incubated at 28 °C, 180 rpm for 36 hr to obtain seeds. The seeds were then inoculated at a ratio of 1:100 into 250 mL Fernbach flasks, each containing 50 mL of 61# medium. After incubating at 28 °C, 180 rpm for 168 hr, the liquid and mycelia were separated by centrifugation, and extracted with ethyl acetate and acetone, respectively. The extracts were combined, dried out with a rotary evaporation apparatus, and dissolved with methanol. The samples were analyzed by using an Agilent 1200 liquid chromatograph system with a Thermo C_18_ column (5 μm, 250 × 4.6 mm), which was equilibrated with 70% solvent A (H_2_O) and 30% solvent B (methanol), developed with a linear gradient (0–25 min, from 30% B to 100% B), then kept at 100% B for 5 min at a flow rate of 1 mL/min, and UV detected at 254 nm. Three independent fermentations were carried out, and compounds isolated from each fermentation were analyzed with three technical replicates.

### Isolation and purification of compounds

The fermentation was carried out for 168 hr and the crude extracts were obtained as described previously. The mixture of compounds was then subjected to silica gel column chromatography, eluting with solvent mixtures of chloroform-methanol (1:0, 40:1, 20:1, 15:1, 9:1 and 0:1, v/v) successively. The chloroform-methanol (40:1, 20:1) eluting fraction was purified by C_18_ chromatography silica gel (Chromatorex) with solvent mixtures of acetonitrile-ddH_2_O (7:3, 8:2, 9:1 and 1:0, v/v) successively. Semi-preparative HPLC was finally used to purify compounds **1**, **2**, and **3** on a LC3000 liquid chromatograph system with a thermo-C_18_ column (5 μm, 250 mm × 10 mm), which was equilibrated with 70% solvent A (acetonitrile) and 30% solvent B (ddH_2_O), developed with a linear gradient (0–15 min, from 70% A to 100% A), and then kept at 100% A for 5 min at a flow rate of 3 mL/min. The elution of compounds was monitored by UV detection at 254 nm. Finally, 9 mg of compound **1** (t_R_ 24.4 min), 12 mg of compound **2** (t_R_ 26.6 min), and 46 mg of compound **3** (t_R_ 28.4 min) were obtained. MS spectra were acquired on an Agilent 1100 instrument, and NMR spectra were acquired on Bruker AV-500 and AV-800 instruments.

### Reverse transcription PCR and real-time PCR analyses of gene expression

Total RNA was isolated from strains cultured for 120 hr in 61# medium with a RNAprep Pure Cell/Bacteria kit (TIANGEN, Beijing), and reverse transcription was carried out by using a PrimeScript RT kit with gDNA Eraser (Takara, DaLian) according to the manufacturer’s instruction. PCR was performed with primers shown in Supplementary Table S4 at the following condition: 94 °C 30 sec, 62 °C 30 sec, and 72 °C 60 sec, for a total of 30 cycles. Samples of each WT and mutant gene pair at certain time point were run on the same gel. The images were captured by using Syngene GeneGenius Gel Imaging System with the same default settings, and the relative expression levels were evaluated by using Genesnap. RNA samples used in real-time PCR were isolated from fermentation cultures incubated for 120 hr. The amplification was carried out on a ABI StepOne Plus system using a SuperReal PreMix Plus kit (TIANGEN, Beijing), with primers listed in Supplementary Table S5 at the following condition: 95 °C 15 min, 1 cycle, followed by 40 cycles of 95 °C 10 sec, and 60 °C 32 sec. Relative standard curve method was used for analyzing the difference in transcription of genes, with 16S rRNA as the internal control. For each assay, RNAs were isolated from three independent fermentation cultures, and the analyses were conducted with three technical replicates.

### Expression and purification of GdmRIII protein

Gene *gdmRIII* was PCR amplified from wild-type *S. autolyticus* with primers 5′-CCGGAATTCATGGTCCCCCGAAGCCCG-3′ and 5′-CCCAAGCTTTCGACTCCCGTCGGGCGGC-3′. The amplicon was cloned into pET29a and then introduced into *E. coli* BL21(DE3) by electroporation. The resultant strain was cultured in LB medium containing kanamycin at 25 °C, 200 rpm until OD_600_ reached around 0.6. IPTG was added at a final concentration of 1 mM, and the culturing was continued under the same conditions for additional 7 hr. Cells were recovered by centrifugation at 4,500 × g and resuspended in PBS Buffer (137 mM NaCl, 2.7 mM KCl, 10 mM Na_2_HPO_4_ and 2 mM KH_2_PO_4_, pH 7.4). After sonication, the proteins were recovered by centrifugation at 26,000 × g for 30 min and filtered with a 0.22 µm Millipore column. The GdmRIII protein was then purified by using a HisTrap HP column (GE Healthcare, Chicago) according to the manufacturer’s instruction. Purified GdmRIII was loaded into a PD-10 desalting column (GE Healthcare) for desalting, and was then concentrated by using a centrifugal filter device. Concentrated GdmRIII was dissolved in solution P (20 mM Tris-Cl, 1 mM EDTA, 200 mM NaCl, 10% glycerol) for further analysis. The GdmRIII was verified by western blot^50^ using anti-His mouse monoclonal antibodys (Trans).

### Electrophoretic mobility shift assay (EMSA)

DNA fragments containing the promoter regions of genes to be analyzed were PCR amplified from the chromosomal DNA of wild-type *S. autolyticus* with the following primers: 5′-GAGAGCGGCTGGCAGCCGGTGTA-3′ and 5′-GCGACGTCCACCGCCGTCTCC-3′ for *gdmM* gene, 5′-GTGCAGGCGCAGTCGGCGTC-3′ and 5′-GCTCACCGACCCCGCGCACGA-3′ for *gdmN* gene, and 5′-AGGAGCTGCCCGACTGGCGGTCCTTCTG-3′ and 5′-AGTCGCTCCAGCCAGTCCTCGATGGCC-3′ for *elaF* gene. Labeling was performed by using an EMSA Probe Biotin Labeling kit (Beyotime, Shanghai), and EMSAs were carried out by using a Chemiluminescent EMSA kit (Beyotime) according to the manufacturer’s instruction, with 1% BSA as the negative control. The composition of the binding buffer was 1 mM DTT, 5% glycerol, 1 mM EDTA, 50 mM NaCl, 10 mM MgCl_2_, 10 mM Tri-HCl (pH7.5), and 0.1 μg/μl poly(dI-dC). DNA at a final concentration of 0.3 nM was used for the EMSAs. For competitive EMSAs, 50-fold specific promoter DNA fragment or 50-fold non-specific DNA fragment was added in the reaction. The non-specific DNA (a non-target sequence that has a similar base component and length to the target sequence) was amplified from the chromosomal DNA of wild-type *S. autolyticus* with primers 5′-GTTGAGGGTCCCCGATCGAGGTCGACG-3′ and 5′-TGACACGGCGCGCTCCGGAAGAGGCG-3′. The concentration of promoter sequences used in the competitive EMSAs was 0.3 nM. The concentrations of GdmRIII in the competitive EMSAs were 1.5 μM for *gdmM*, *gdmN* and 1.2 μM for *elaF*. The binding of the GdmRIII to the DNA fragments was analyzed by 6% PAGE. Each EMSA was repeated three times.

### Mapping of the binding sites of GdmRIII by DNase I footprinting

The promoter region of *elaF* that contains the probable binding sites of GdmRIII was amplified from the chromosomal DNA of wild-type *S. autolyticus* with primers 5′-AGGAGCTGCCCGACTGGCGGTCCTTCTG-3′ and 5′-AGTCGCTCCAGCCAGTCCTCGATGGCC-3′, and then cloned into pEASYT1. The resultant plasmids were used as the templates for preparing fluorescent FAM labeled probes. The probes were amplified with Dpx DNA polymerase (TOLO Biotech, Shanghai), by using primers of M13F-47 (FAM) (5′-CGCCAGGGTTTTCCCAGTCACGAC-3′) and M13R-48 (5′-AGCGGATAACAATTTCACACAGGA-3′). The FAM-labeled probes were purified by using a Wizard^®^ SV Gel and PCR Clean-Up System (Promega, Madison) according to the manufacturer’s instruction, and quantified with NanoDrop 2000C. DNase I footprinting assays were performed as described^52^, except that 400 ng of probes and 1.6 µM of GdmRIII proteins were used in the assays.

### Data availability

The datasets generated during and/or analysed during the current study are available from the corresponding author on reasonable request.

## Electronic supplementary material

Supplementary Information
