# Supplementary material for: GdmRIII, a TetR Family Transcriptional Regulator, Controls Geldanamycin and Elaiophylin Biosynthesis in Streptomyces autolyticus CGMCC0516
Source: Sci Rep. 2017 Jul 6;7:4803. doi: 10.1038/s41598-017-05073-x (PMC5500506; doi:10.1038/s41598-017-05073-x)
Supplement: Supplementary file 1 — Supplementary Information [file 41598_2017_5073_MOESM1_ESM.pdf]

GdmRIII, a TetR Family Transcriptional Regulator, Controls Geldanamycin and Elaiophylin Biosynthesis in *Streptomyces autolyticus* CGMCC0516

MingXing Jiang+, Min Yin+, ShaoHua Wu, XiuLin Han, KaiYan Ji, MengLiang Wen* and Tao Lu*

Supplementary Information

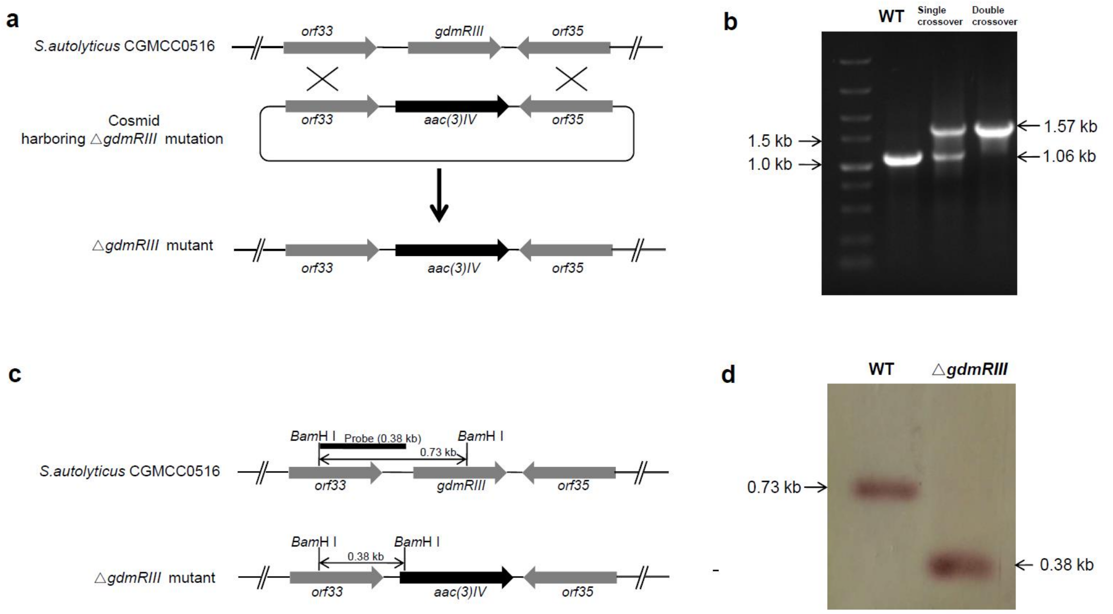

Fig. S1 Construction and verification of the Δ*gdmRIII* mutant.

(a) Schematic representation of the construction of the Δ*gdmRIII* mutant; (b) verification of the Δ*gdmRIII* mutant by PCR amplification (the sizes of the PCR products from the wild-type strain, the single-crossover mutant, and the double-crossover mutant were 1.06 kb, 1.06 kb and 1.57 kb, 1.57 kb, respectively); (c) schematic representation of the Southern blot verification of the Δ*gdmRIII* mutant; (d) verification of the Δ*gdmRIII* by Southern blot (the sizes of the hybridized DNA fragments from the wild-type strain and the mutant were 0.73 kb and 0.38 kb, respectively).

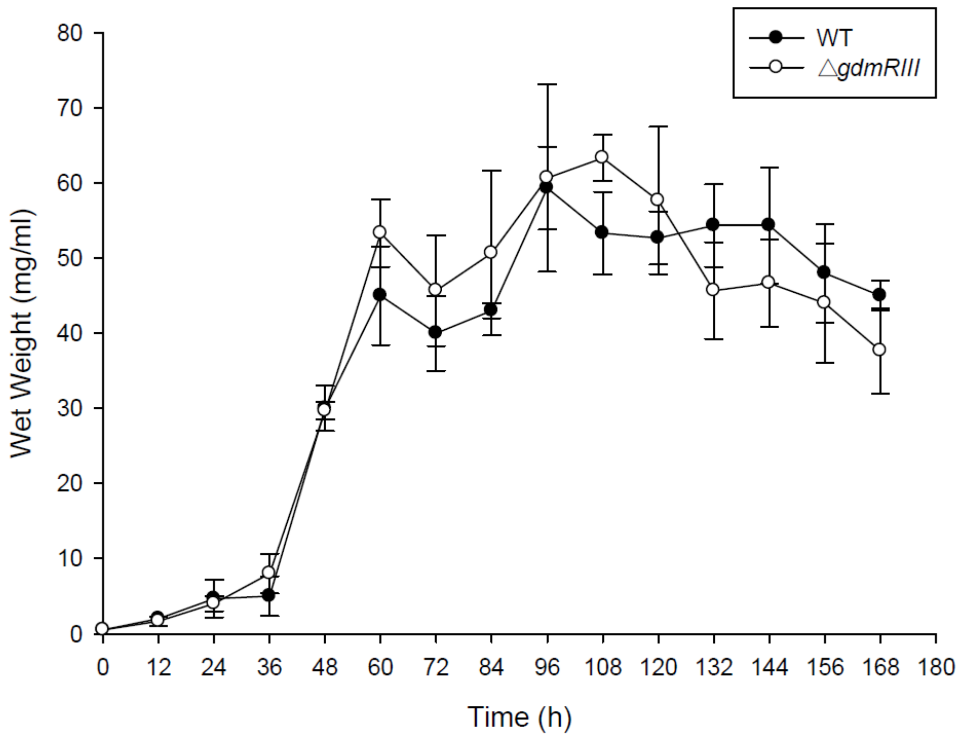

Fig. S2 The growth of the *S. autolyticus* wild-type strain and the ∆*gdmRIII* mutant. Values are the average of three independent experiments. Error bars indicate the standard deviation of means.

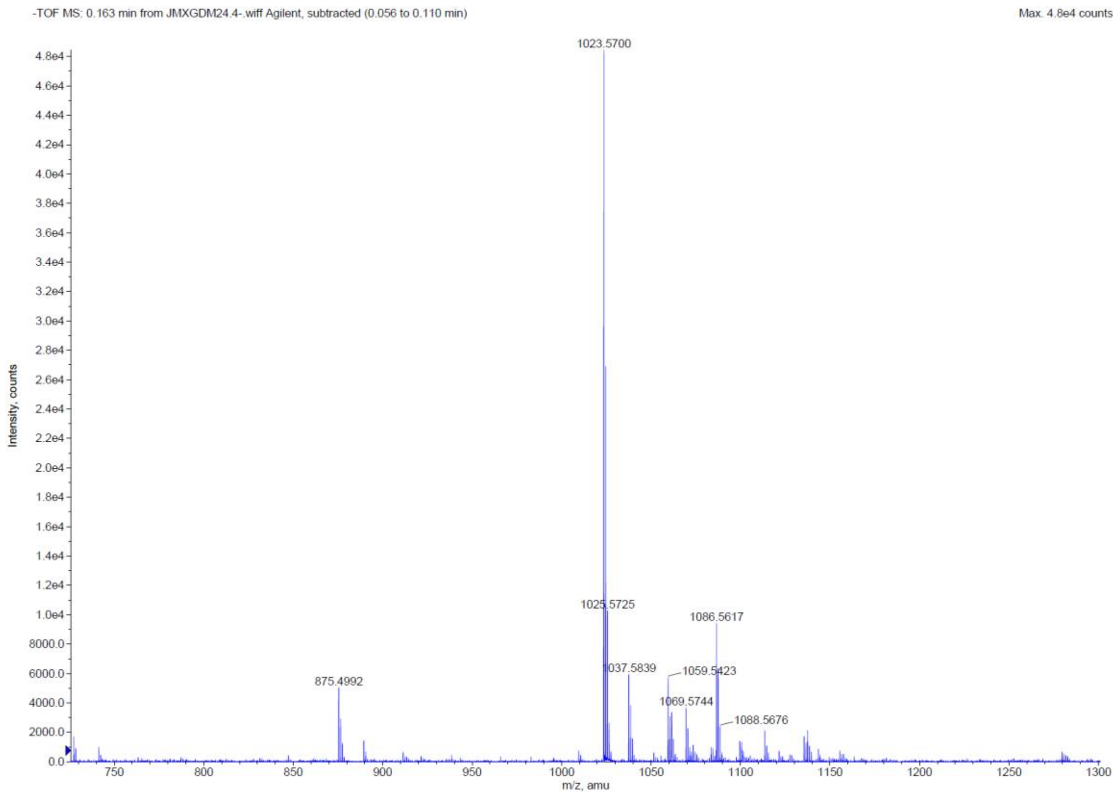

Fig. S3 The HRESI-MS(–) of Compound 1.

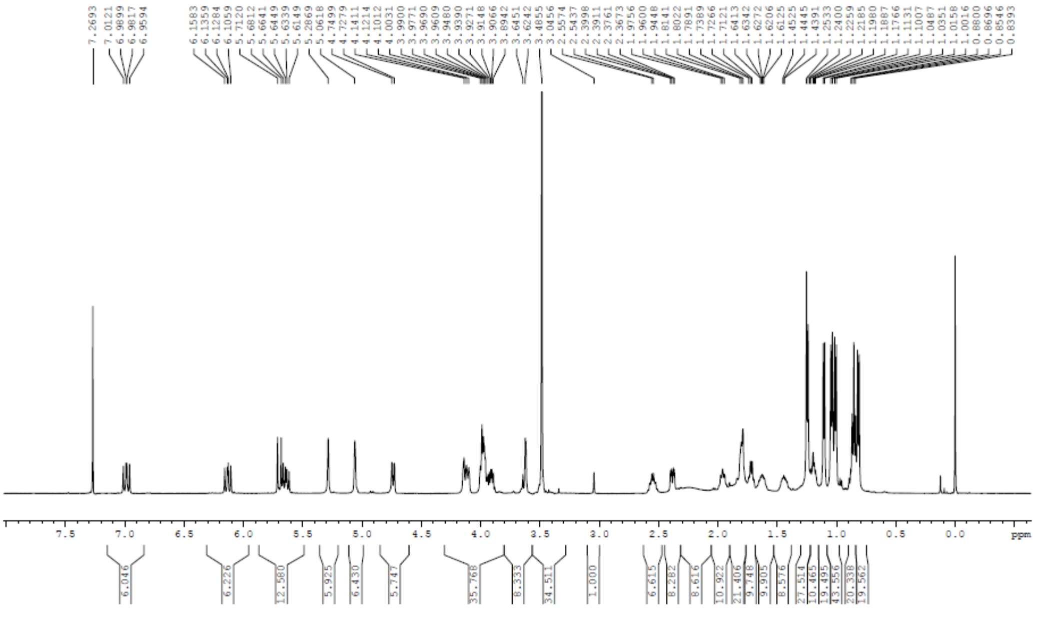

Fig. S4 The ^{1}H-NMR Spectrum of Compound 1 in $CDCl_3$.

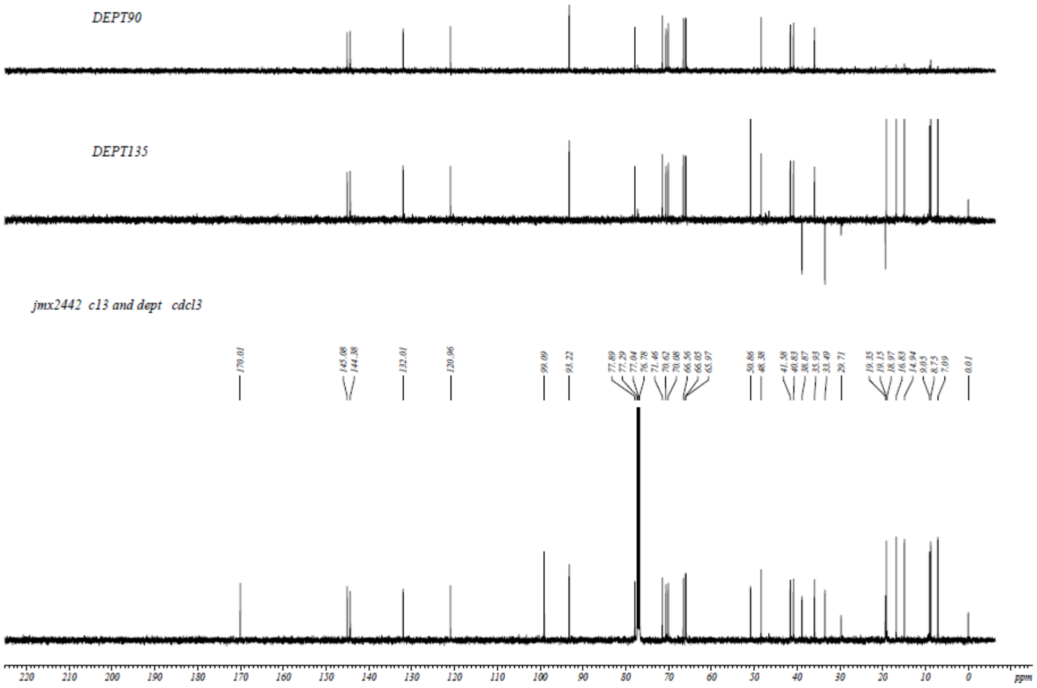

Fig. S5 The ^{13}C-NMR and DEPT Spectrum of Compound 1 in $CDCl_3$.

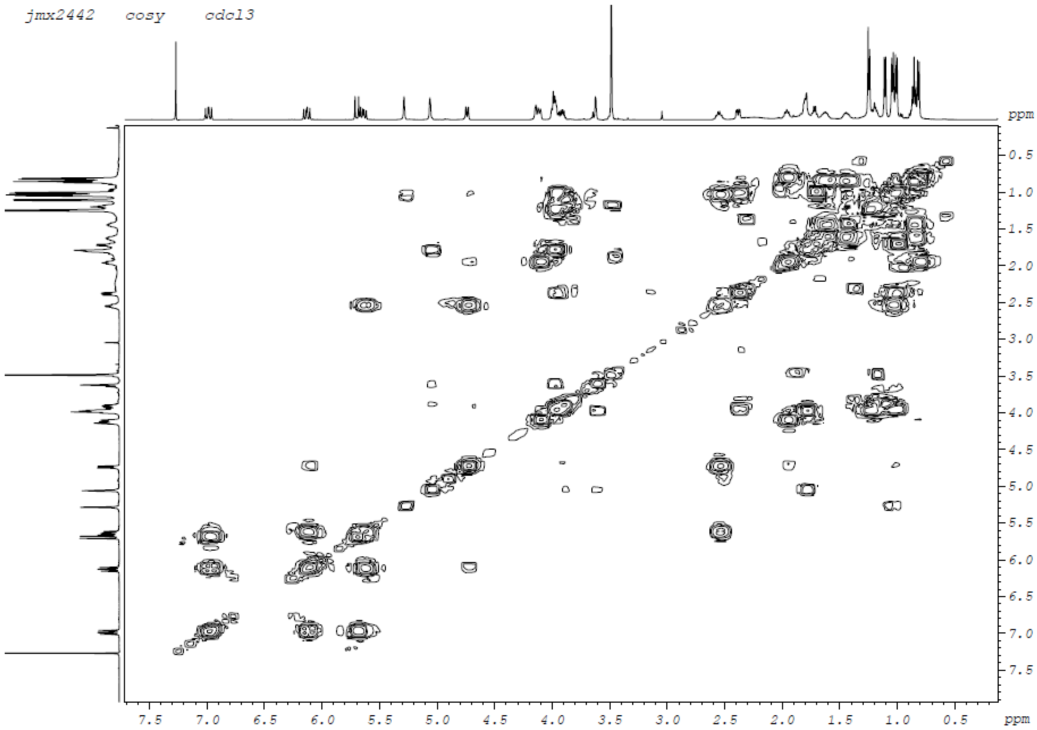

Fig. S6 The ^{1}H-^{1}H COSY Spectrum of Compound 1 in $CDCl_3$.

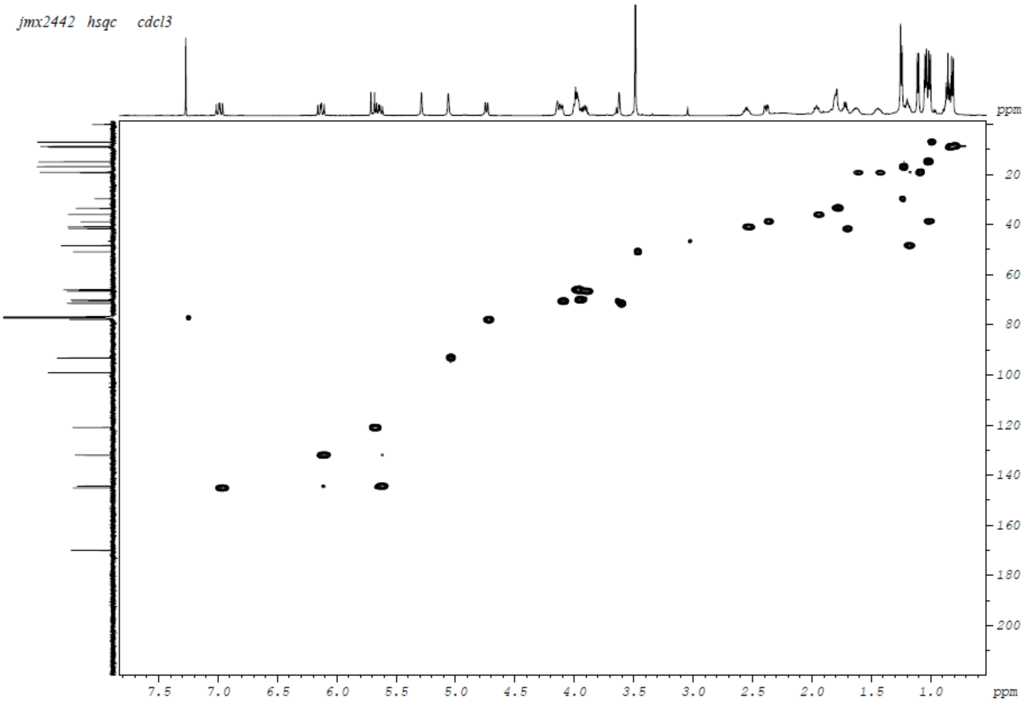

Fig. S7 The HSQC Spectrum of Compound 1 in $CDCl_3$.

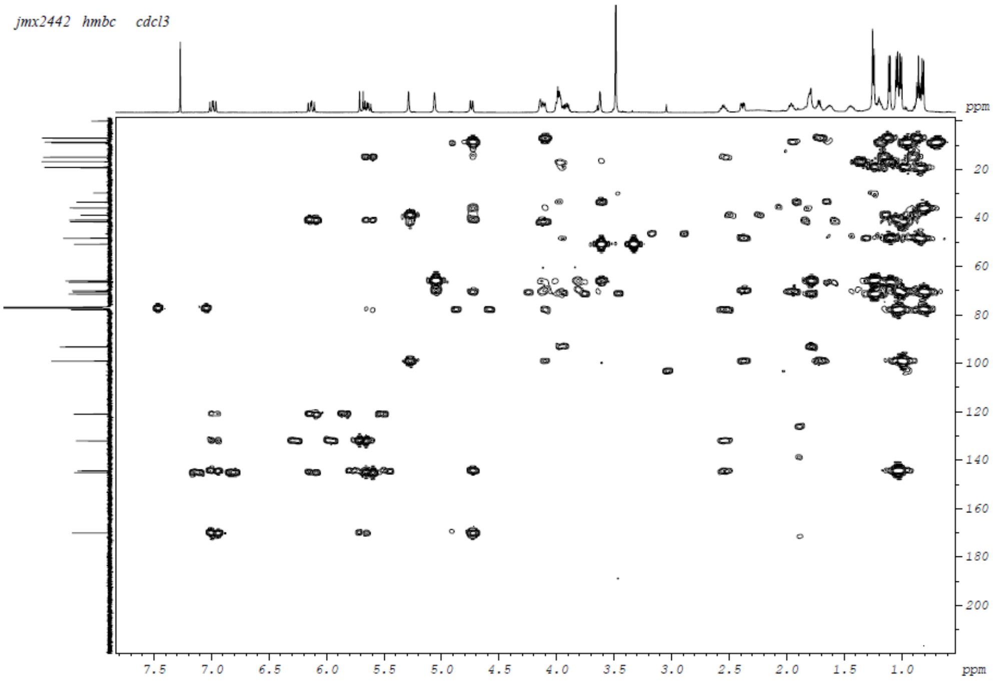

Fig. S8 The HMBC Spectrum of Compound 1 in $CDCl_3$.

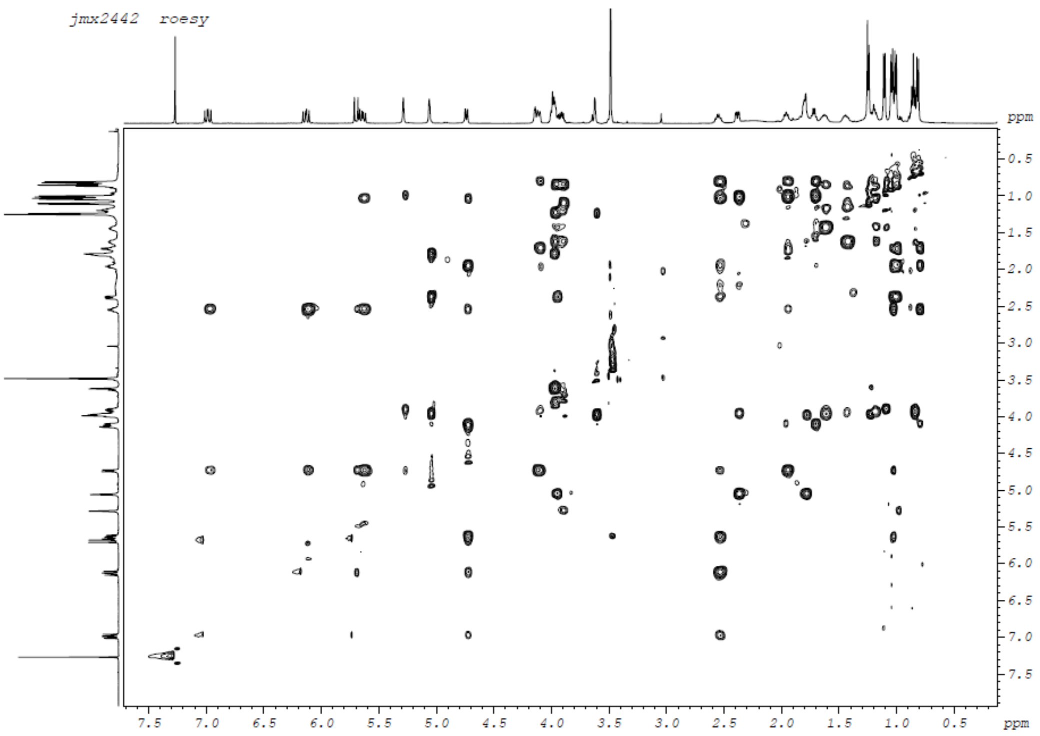

Fig. S9 The ROSEY Spectrum of Compound 1 in $CDCl_3$.

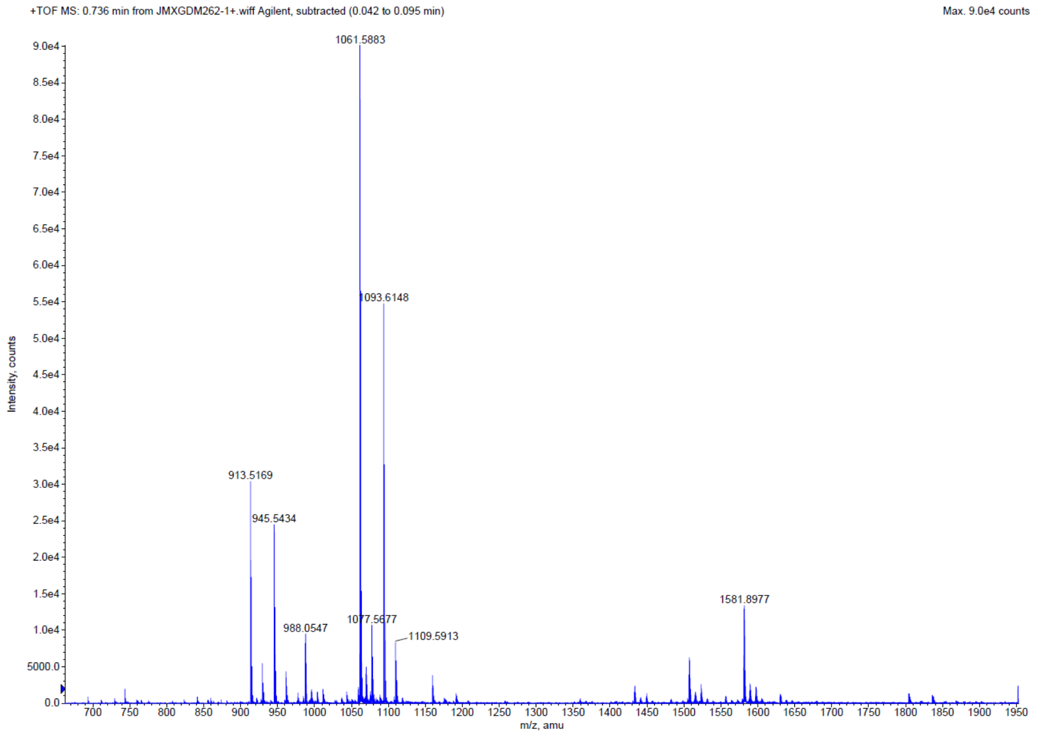

Fig. S10 The HRESI-MS(+) of Compound 2.

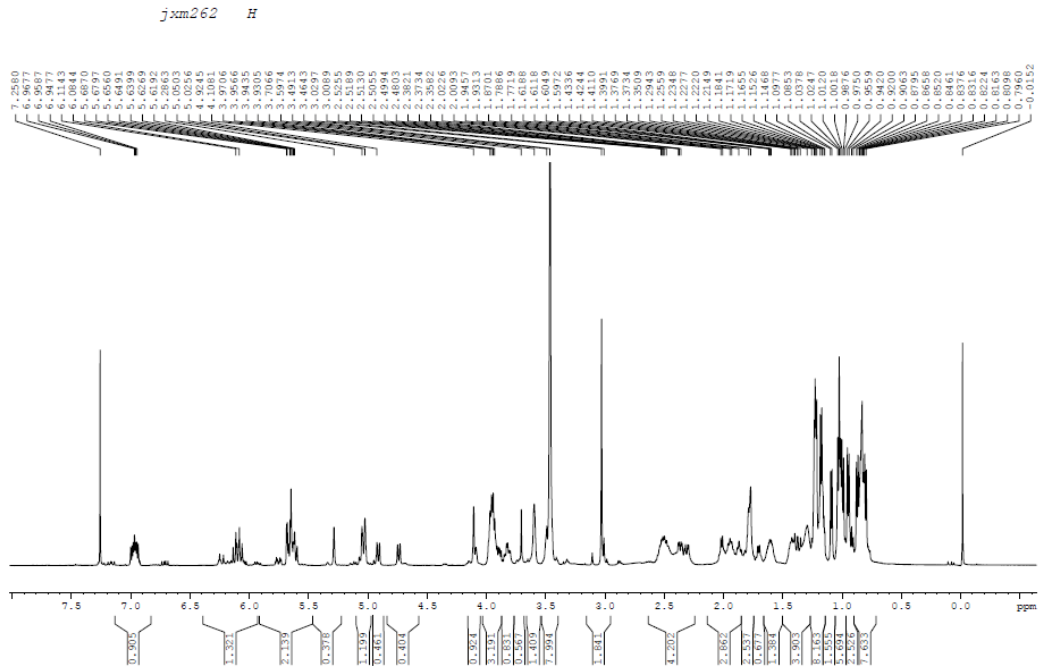

Fig. S11 The ^{1}H-NMR Spectrum of Compound 2 in $CDCl_3$.

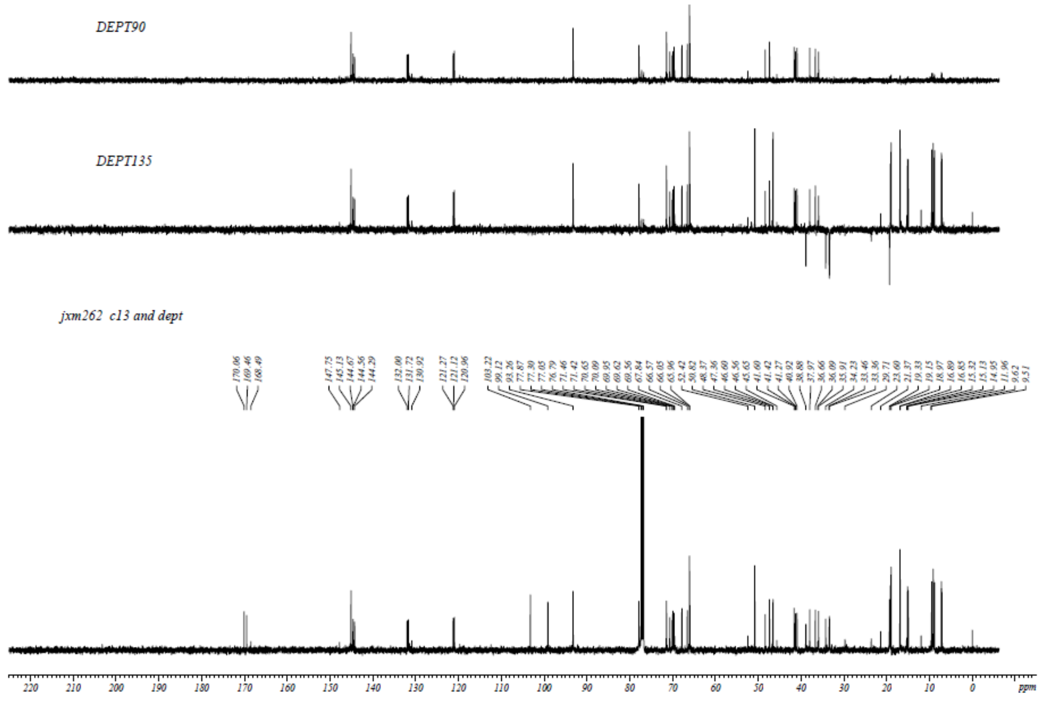

Fig. S12 The ^{13}C-NMR and DEPT Spectrum of Compound 2 in $CDCl_3$.

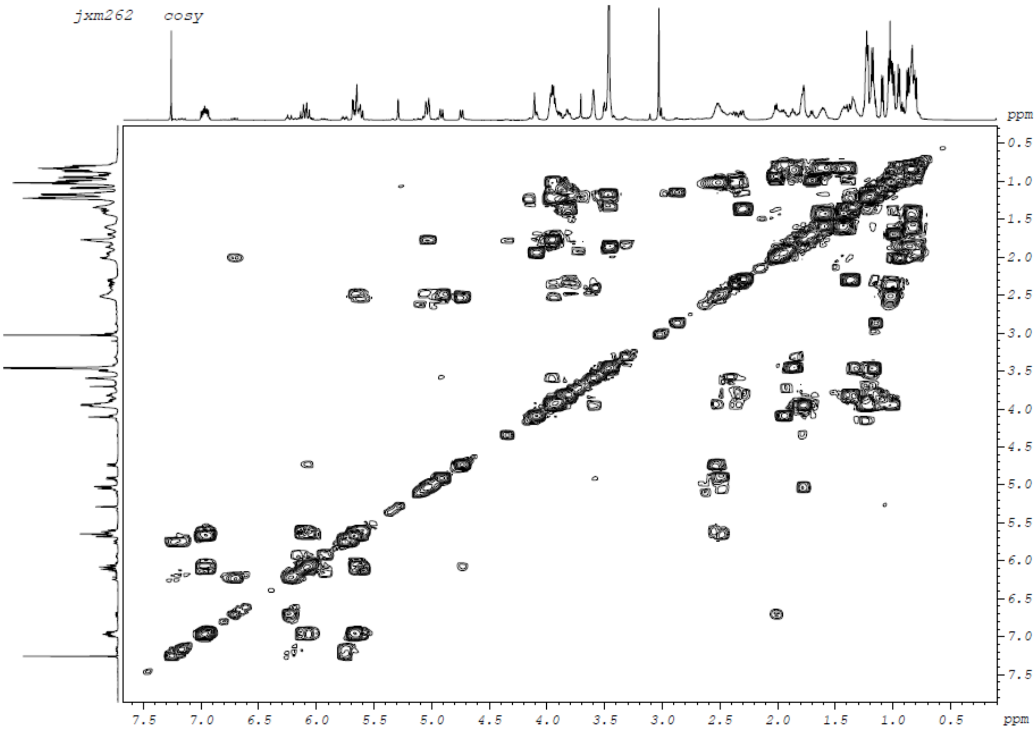

Fig. S13 The ^{1}H-^{1}H COSY Spectrum of Compound 2 in $CDCl_3$.

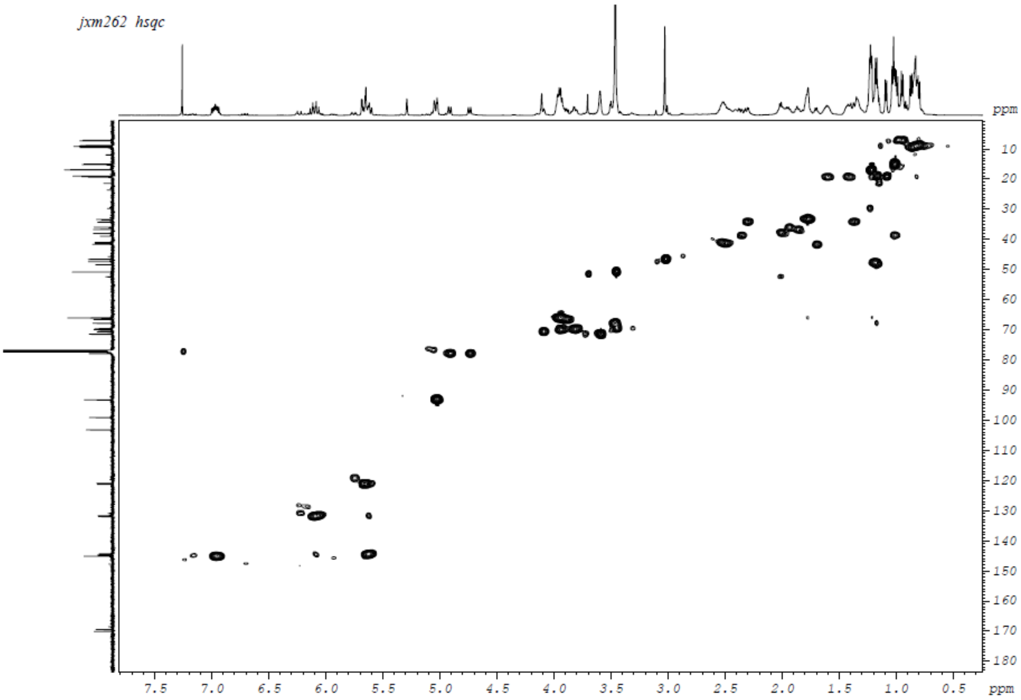

Fig. S14 The HSQC Spectrum of Compound 2 in $CDCl_3$.

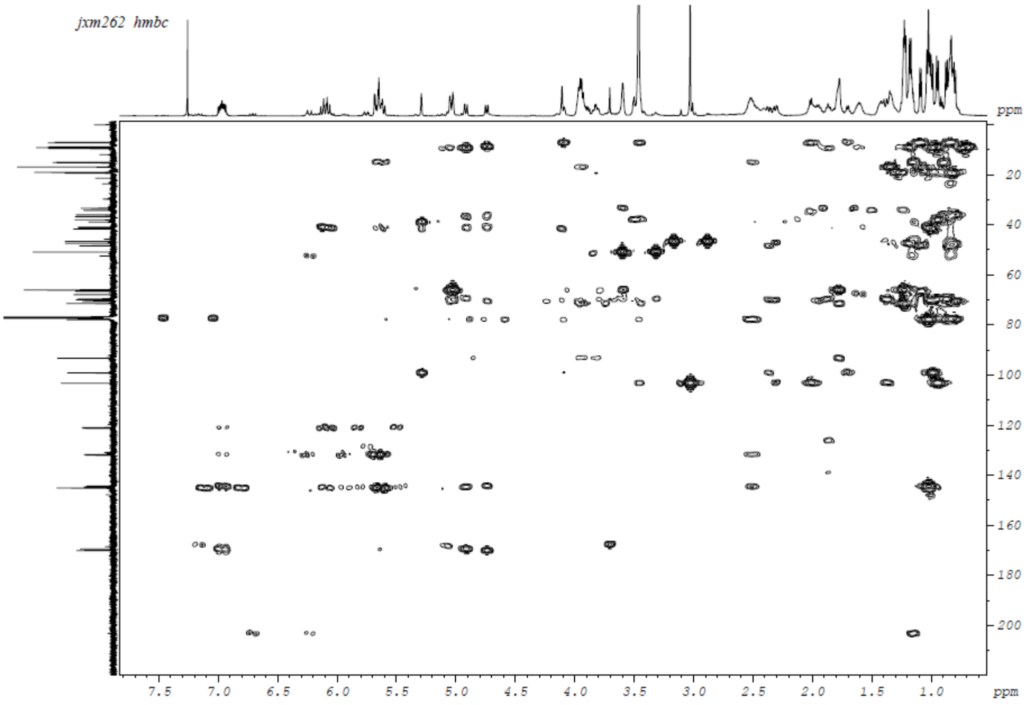

Fig. S15 The HMBC Spectrum of Compound 2 in $CDCl_3$.

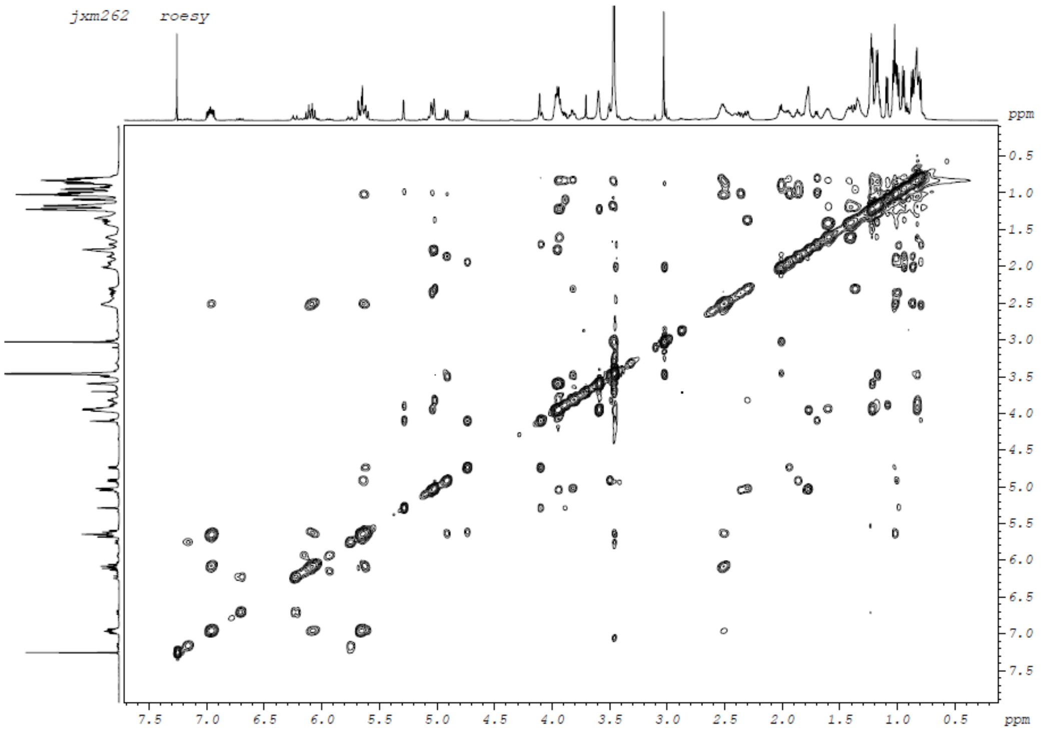

Fig. S16 The ROSEY Spectrum of Compound 2 in $CDCl_3$.

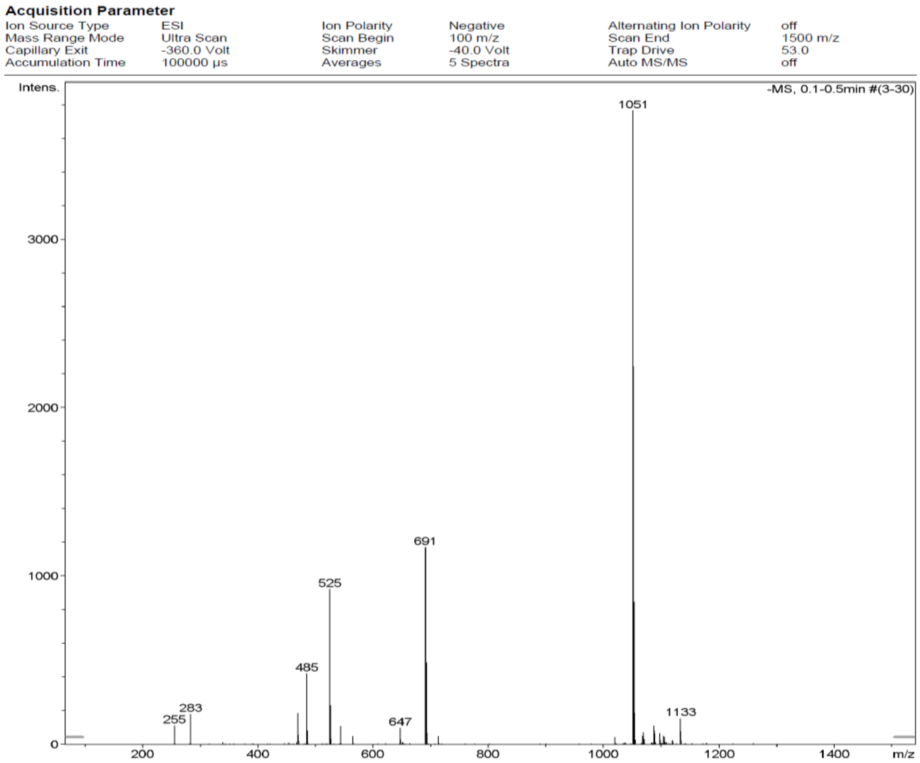

Fig. S17 The HRESI-MS(–) of Compound 3.

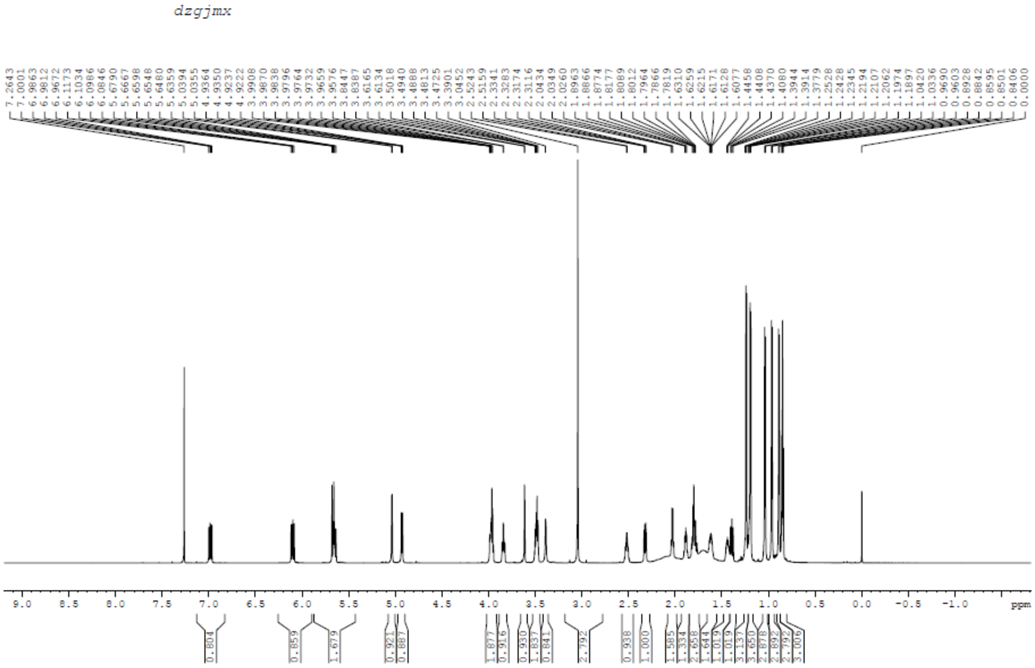

Fig. S18 The ^{1}H-NMR Spectrum of Compound 3 in $CDCl_3$.

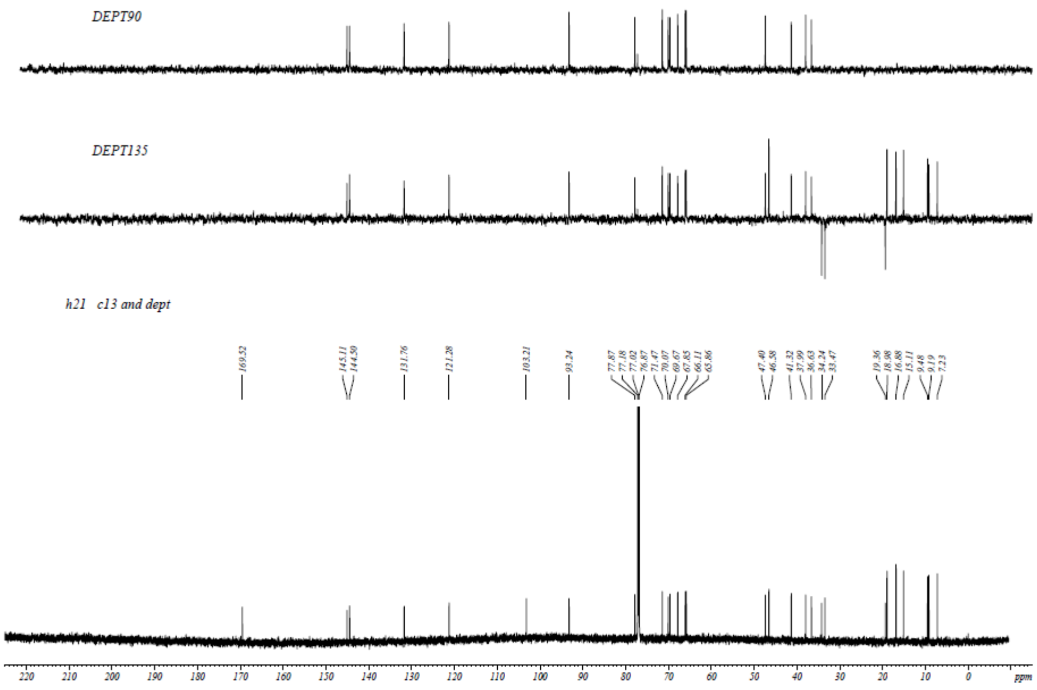

Fig. S19 The ^{13}C-NMR and DEPT Spectrum of Compound 3 in $CDCl_3$.

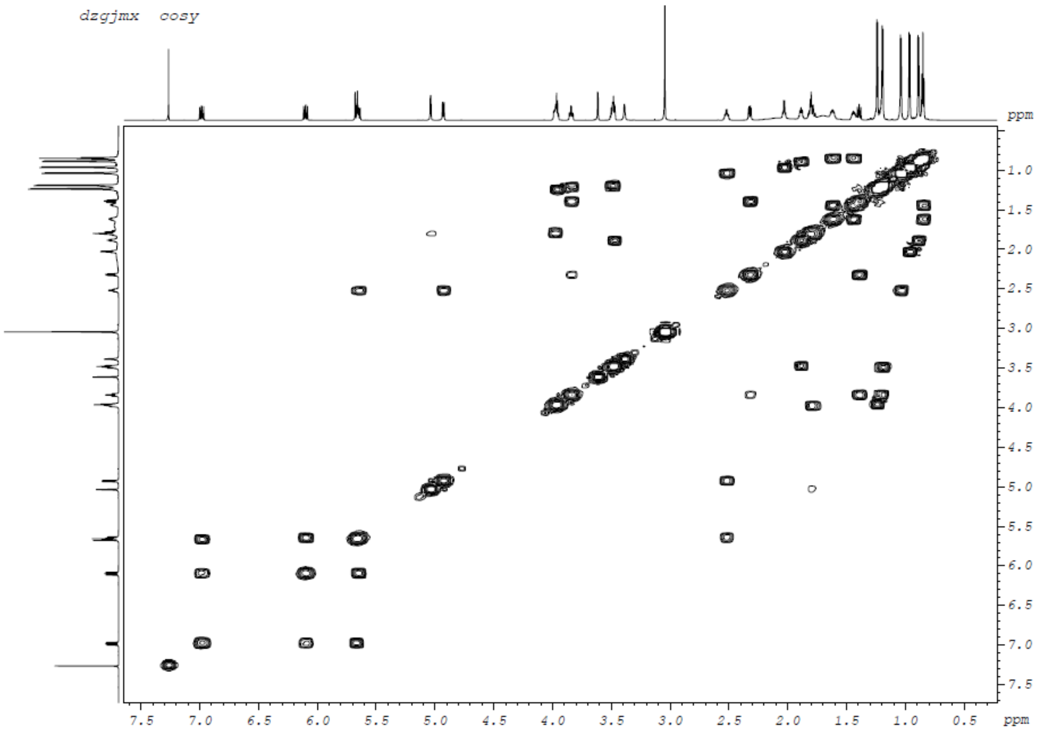

Fig. S20 The ^{1}H-^{1}H COSY Spectrum of Compound 3 in $CDCl_3$.

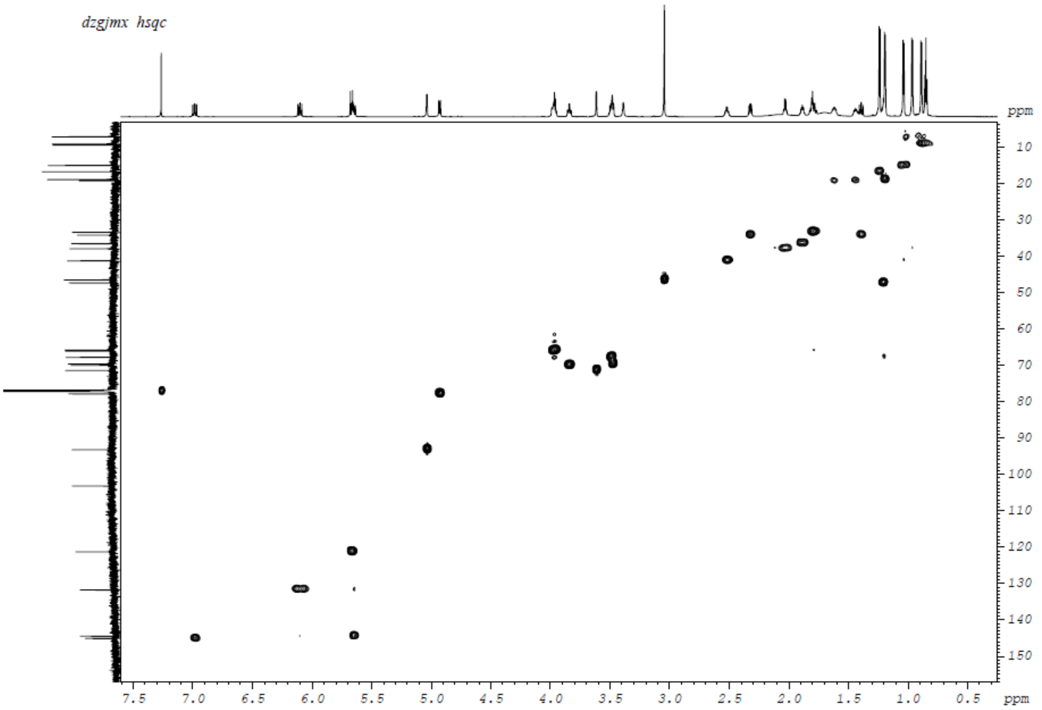

Fig. S21 The HSQC Spectrum of Compound 3 in $CDCl_3$.

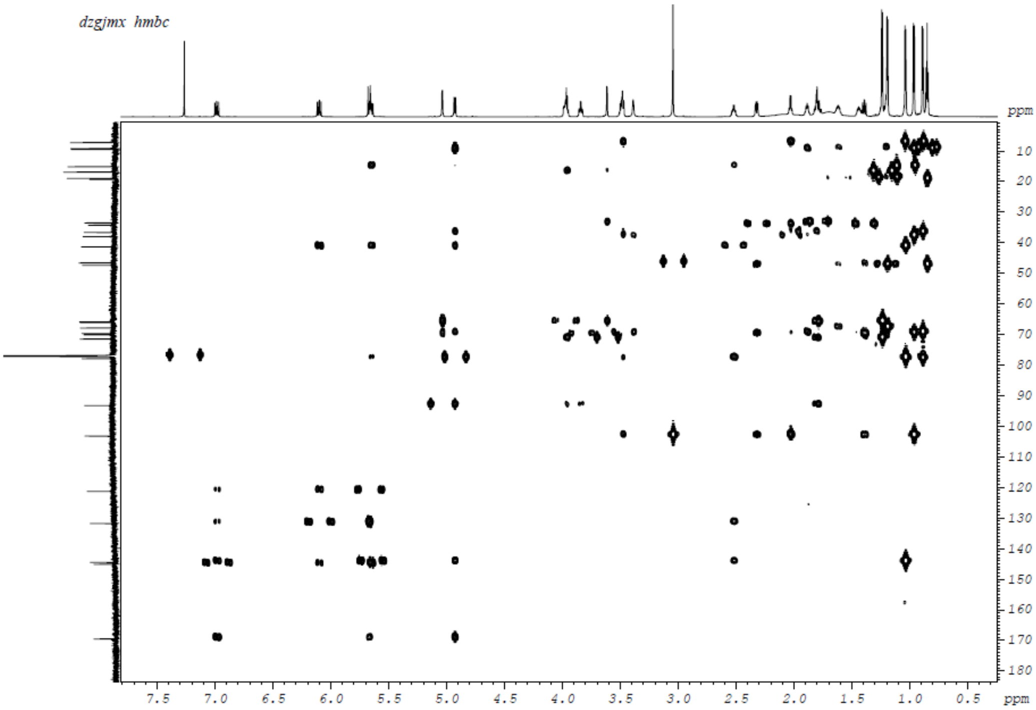

Fig. S22 The HMBC Spectrum of Compound 3 in $CDCl_3$.

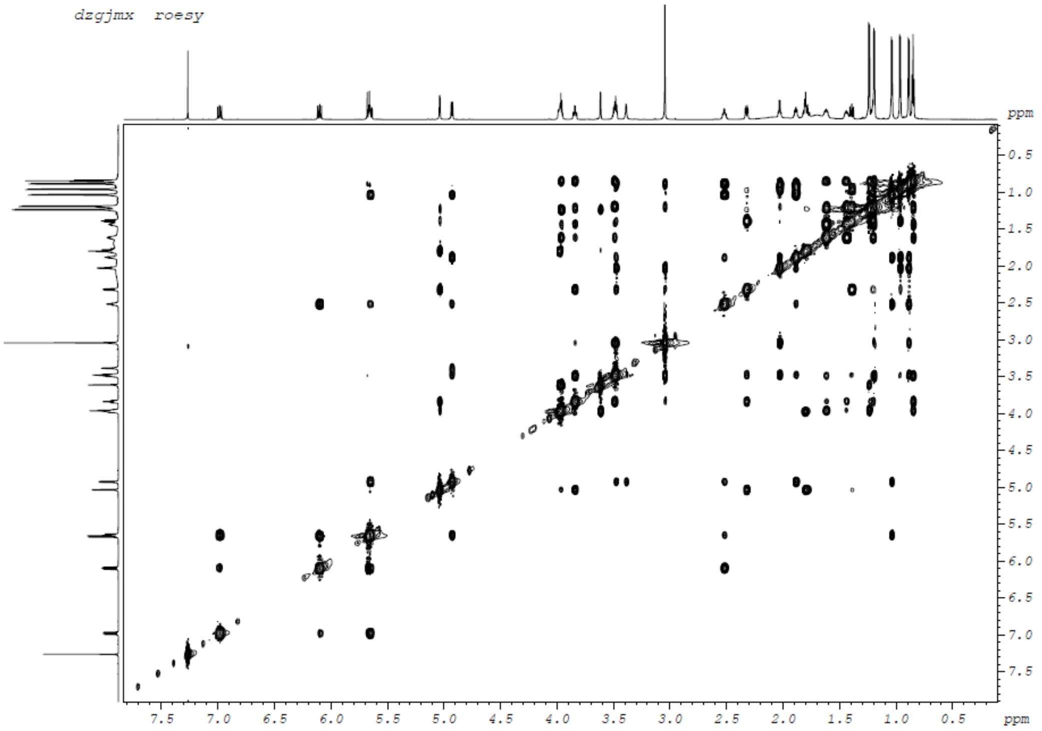

Fig. S23 The ROSEY Spectrum of Compound 3 in CDCl₃.

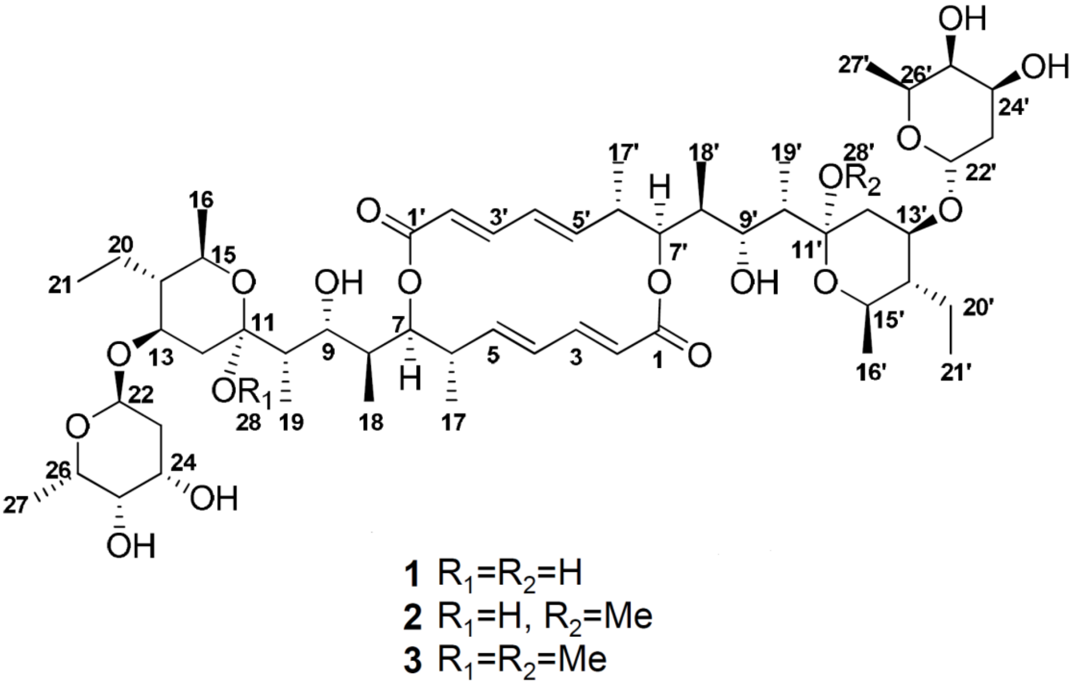

1 R$_1$=R$_2$=H
2 R$_1$=H, R$_2$=Me
3 R$_1$=R$_2$=Me

Fig. S24 The chemical structure of compounds 1, 2, and 3.

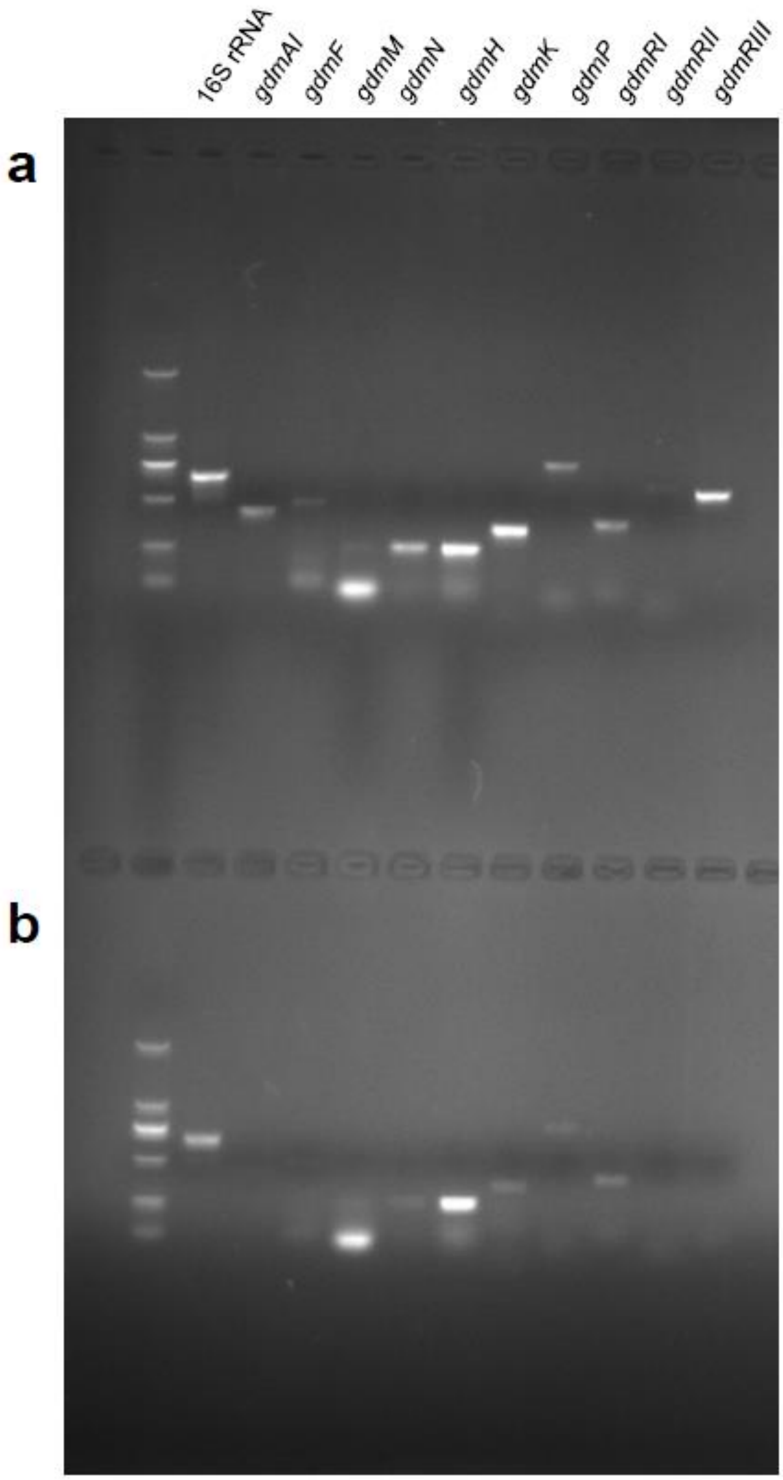

Fig. S25 RT-PCR Analysis of the transcriptional levels of genes in the geldanamycin biosynthetic gene cluster.

(a) The wild-type strain; (b) the Δ*gdmRIII* mutant. The experiment was repeated three times, and the representative image is shown.

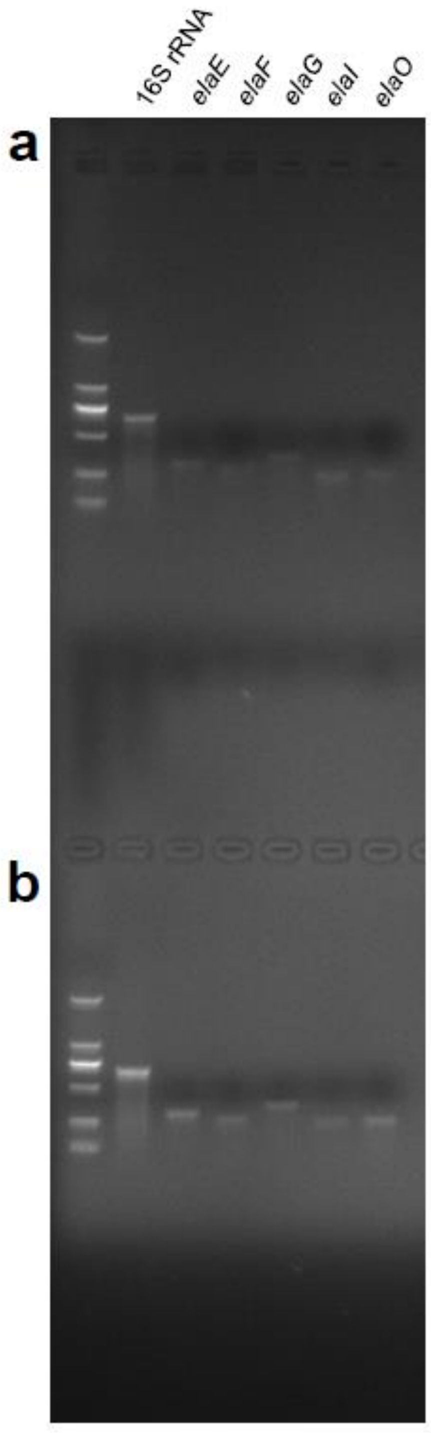

Fig. S26 RT-PCR Analysis of the transcriptional levels of genes in the elaiophylin biosynthetic gene cluster.

(a) The wild-type strain; (b) the Δ*gdmRIII* mutant. The experiment was repeated three times, and the representative image is shown.

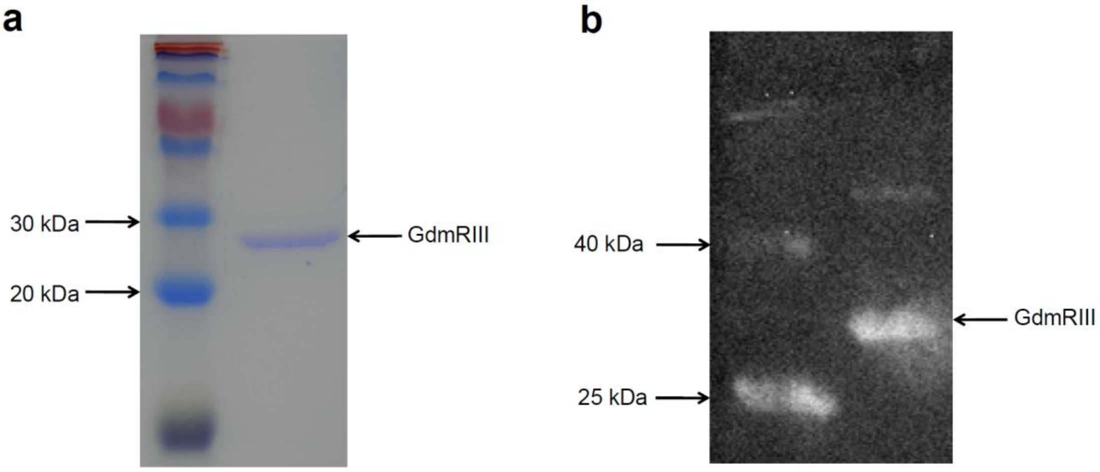

Fig. S27 Analysis of the purified GdmRIII protein by using SDS-PAGE (a) and Western blot (b).

Table S1 ^{1}H (500 MHz) and ^{13}C NMR (125 MHz) Data for Compounds 1 in $CDCl_3$.

No.	δ_H, mult. (J in Hz)	δ_C
1		170.0 s
2	5.70 (d, 15.2)	121.0 d
3	6.99 (dd, 15.2, 11.2)	145.1 d
4	6.13 (dd, 15.2, 11.2)	132.0 d
5	5.64 (dd, 15.2, 9.6)	144.4 d
6	1.72 (q, 6.8)	41.6 d
7	4.74 (dd, 10.0, 0.8)	77.9 d
8	1.96 (m)	35.9 d
9	3.96 (m)	70.1 d
10	2.55 (m)	40.8 d
11		99.1 s
12	1.02 (dd, 11.8, 4.3)	38.9 t
	2.38 (dd, 11.8, 4.3)	
13	4.13 (m)	70.6 d
14	1.19 (m)	48.4 d
15	3.91 (m)	66.6 d
16	1.11 (d, 6.3)	19.2 q
17	1.04 (d, 6.8)	14.9 q
18	0.86 (d, 7.6)	9.0 q
19	1.01 (d, 7.2)	7.1 q
20	1.44 (ddd, 14.1, 7.0, 3.8)	19.4 t
	1.63 (ddd, 14.1, 7.0, 3.8)	
21	0.82 (d, 7.0)	8.7 q
22	5.06 (br s)	93.2 d
23	1.80 (m)	33.5 t
24	3.97 (brs)	66.0 d
25	3.62 (brs)	71.5 d
26	3.99 (m)	66.1 d
27	1.25 (d, 6.6)	16.8 q

Table S2 ^{1}H (500 MHz) and ^{13}C NMR (125 MHz) Data for Compounds 2 in $CDCl_3$.

No.	δ_H, mult. (J in Hz)	δ_C	No.	δ_H, mult. (J in Hz)	δ_C
1		170.1 s	1'		169.5 s
2	5.67 (d, 15.4)	121.3 d	2'	5.67 (d, 15.4)	121.0 d
3	6.97 (dd, 15.4, 10.9)	145.1 d	3'	6.97 (dd, 15.4, 10.9)	145.1 d
4	6.08 (dd, 15.3, 10.9)	131.7 d	4'	6.12 (dd, 15.3, 10.9)	132.0 d
5	5.62 (dd, 15.3, 9.9)	144.3 d	5'	5.62 (dd, 15.3, 9.9)	144.7 d
6	2.49 (m)	40.9 d	6'	2.53 (m)	41.3 d
7	4.74 (dd, 10.1, 0.8)	77.9 d	7'	4.91 (dd, 10.1, 1.0)	77.9 d
8	1.95 (m)	35.9 d	8'	1.87 (m)	36.7 d
9	3.60 (m)	71.4 d	9'	3.60 (m)	71.5 d
10	1.70 (m)	41.6 d	10'	2.02 (m)	38.0 d
11		99.1 s	11'		103.2 s
12	1.02 (m)<br>2.37 (dd, 11.9, 4.3)	38.9 t	12'	1.38 (m)<br>2.31 (dd, 13.3, 4.4)	34.2 t
13	3.95 (m)	70.0 d	13'	3.83 (td, 10.5, 4.9)	69.6 d
14	1.20 (m)	47.4 d	14'	1.17 (m)	48.4 d
15	3.96 (m)	66.0 d	15'	3.96 (m)	66.6 d
16	1.18 (d, 6.1)	19.0 q	16'	1.09 (d, 6.3)	19.2 q
17	1.03 (d, 6.6)	14.9 q	17'	1.03 (d, 6.6)	15.1 q
18	0.80 (d, 6.9)	8.8 q	18'	0.82 (d, 6.8)	9.1 q
19	0.95 (d, 7.0)	7.1 q	19'	0.99 (d, 7.0)	7.2 q
20	1.43 (m)	19.3 t	20'	1.61 (m)	19.4 t
21	0.83 (d, 6.5)	9.0 q	21'	0.85 (t, 6.6)	9.5 q
22	5.04 (m)	93.2 d	22'	5.04 (m)	93.3 d
23	1.78 (m)	33.4 t	23'	1.78 (m)	33.5 t
24	3.96 (m)	66.1 d	24'	3.50 (m)	67.8 d
25	3.96 (m)	70.1 d	25'	4.11 (m)	70.7 d
26	3.96 (m)	66.0 d	26'	3.96 (m)	66.1 d
27	1.22 (d, 6.6)	16.8 q	27'	1.23 (d, 6.5)	16.9 q
11-OH	5.29 (br s)		28'	3.03 (s)	46.6 q

Table S3 ^{1}H (800 MHz) and ^{13}C NMR (200 MHz) Data for Compounds 3 in $CDCl_3$.

No.	δ_H, mult. (*J* in Hz)	δ_C
1		169.5 s
2	5.67 (d, 15.4)	121.3 d
3	6.98 (dd, 15.4, 11.2)	145.1 d
4	6.10 (dd, 14.9, 11.2)	131.7 d
5	5.65 (dd, 14.9, 9.6)	144.5 d
6	2.52 (m)	41.3 d
7	4.93 (dd, 10.4, 1.8)	77.9 d
8	1.89 (m)	36.6 d
9	3.48 (m)	69.6 d
10	2.03 (m)	38.0 d
11		103.2 s
12	1.39 (dd, 13.4, 11.0)<br>2.32 (dd, 13.4, 4.6)	34.2 t
13	3.84 (td, 10.6)	70.1 d
14	1.21 (m)	47.4 d
15	3.48 (m)	69.6 d
16	1.19 (d, 6.2)	19.0 q
17	1.04 (d, 6.7)	15.1 q
18	0.89 (d, 6.9)	9.5 q
19	0.96 (d, 6.9)	7.2 q
20	1.44 (ddd, 14.4, 7.4, 4.1)<br>1.62 (ddd, 14.4, 7.4, 4.1)	19.3 t
21	0.85 (t, 7.6)	9.2 q
22	5.04 (d, 2.9)	93.2 d
23	1.79 (m)	33.5 t
24	3.96 (m)	65.8 d
25	3.62 (m)	71.5 d
26	3.98 (m)	66.1 d
27	1.24 (d, 6.5)	16.9 q
28	3.05 (s)	46.6 q

Table S4 Primers used for the reverse transcription PCR analyses.

Gene	Forward primer (5′-3′)	Reverse primer (5′-3′)
16S rRNA	CTACGGGAGGCAGCAGT	TCCGGTGTATGTCAAGC
gdmAI	CAACGGGTGATACGACAAGCC	CGTACCGCTGATACCGAAGGA
gdmF	CCTGGTGTTCGAGCATGTGG	TCTTTCCGTTGTCCGTGGC
gdmM	GCCCAAGACCGATGTGCTGA	GACCACCCACGAAGCGATGAG
gdmN	TCGTACTCGCCGTTGTCCTG	ACACCGTCCTCAACCACCTCTA
gdmH	GGAACCCCGACTGGTACATGG	CGTCTGGGACCTCGACAACA
gdmK	TCGGGATGGAGGAGGTGTTCG	ACCGGCTCATGGTGCTGGGT
gdmP	AAGCCGAACCCGATGTGC	CACGAGACCACGGCCAACA
gdmRI	CAGCATCCCGCTCACCAT	TGTCGCACTCCAACCATTTCT
gdmRII	CGCCGTTCGGAGTGCGTCAA	CGTGCCCAACGCCATGTTC
gdmRIII	CCGTCGGTCAATGAGGAGTTGC	GCTCCCAGTCCAGTCCATAGCG
elaE	GGCCGTATCGCCTACACCTTC	CATCCGACAGCCGCTCCAC
elaF	GCTGTTCGGCCACAGCATG	CCTTGTAGTCGCTGCGGATGG
elaG	GTCGGCAAGGACCACAATCTC	CCAGGAACACCTCACGCATC
elaI	CTGCTGGTCACCCACTTCATG	CCGTCACCTGGGACTTGGA
elaO	ACAAGGTGCCGCATGTGCT	CGGTTGAAGGCGTGGAAGAA

Table S5 Primers used for the real-time PCR analyses.

Gene	Forward primer (5′-3′)	Reverse primer (5′-3′)
16S rRNA	TGGGACAAGCCCTGGAA	CCATCACCCCACCAACAA
gdmAI	AACATCCCGAGCGACCAT	GCGGAGGAGTAGACGACGAA
gdmF	GCACCAACTCTGGACACCCTC	GTCACCACATGCTCGAACACC
gdmM	GCCCTGGAGTCCGCACTGATC	GACCACCCACGAAGCGATGAG
gdmN	TTGAGGCTGGAGTTGTGGGC	CAGGAGGCGGTCGAGAAGATC
gdmH	GCACCGATTCCGACTTGGG	TCGTCTGGGACCTCGACAACA
gdmK	TGCCCTCCTGCACCACACGG	CGAACACCTCCTCCATCCC
gdmP	AGCCGAACCCGATGTGC	CCAACGCGGTGGACGAA
gdmRI	TTCCGCACTCGTCCATC	TGCTCTACCAATCCGTCAA
gdmRII	CGCTCGCAGGGAGAACA	ATCCGATGGACACGGTATTG
elaE	GACACCTACGTGCCCAAGAGC	TGGTGACGAACACGCCCTC
elaF	TGCCCGTGGCGTTCTTC	TTGTAGTCGCTGCGGATGG
elaG	CCCGCCAACACCCGTATCG	GATGGTGGGAATGAACTGC
elaI	GCAAGGGCAATGTCGTCT	AGTTGGCGGGCTTGGAGC
elaO	CAACCGCTATCTGGTCGTG	TTGACGTAATGGCTGTGGC
